# Air-Dried Brown Seaweed, *Ascophyllum nodosum*, Alters the Rumen Microbiome in a Manner That Changes Rumen Fermentation Profiles and Lowers the Prevalence of Foodborne Pathogens

**DOI:** 10.1128/mSphere.00017-18

**Published:** 2018-01-31

**Authors:** Mi Zhou, Martin Hünerberg, Yanhong Chen, Tim Reuter, Tim A. McAllister, Franklin Evans, Alan T. Critchley, Le Luo Guan

**Affiliations:** a Department of Agricultural, Food and Nutritional Science, University of Alberta, Edmonton, Alberta, Canada; b Lethbridge Research Centre, Agriculture and Agri-Food Canada, Lethbridge, Alberta, Canada; c Alberta Agriculture and Rural Development, Agriculture Centre, Lethbridge, Alberta, Canada; d Acadian Seaplants Ltd., Dartmouth, Nova Scotia, Canada; University of Wisconsin—Madison

**Keywords:** *Ascophyllum nodosum*, *Escherichia coli*, Tasco, ram, rumen microbiome, seaweed

## Abstract

The use of Tasco (air-dried *Ascophyllum nodosum*) as a feed supplement for ruminants has been reported to affect rumen fermentation and reduce *Escherichia coli* O157:H7 shedding in feces, but the mode of action behind this phenomenon is unclear. In this study, the effects of four Tasco levels (0, 1, 3, and 5%) on rumen microbiota and rumen/fecal *E. coli* O serogroups in rams were investigated. Rumen total bacteria and archaea were linearly reduced (*P* < 0.001) and protozoa were linearly increased (*P* < 0.001) by increasing levels of Tasco. The relative abundances of seven bacterial species and one protozoal species differed among Tasco levels. With Tasco, 14 predicted metabolic pathways were enriched while only 3 were suppressed. A lower ruminal butyrate concentration is possibly associated with enrichment of the “butanoate metabolism” pathway in Tasco-fed rams. The ruminal total *E. coli* population was linearly reduced (*P* < 0.001) by Tasco. Supplementation with Tasco only completely eliminated O121 in the rumen and feces, and higher levels of Tasco (3 and 5%) reduced fecal shedding of serogroups O45, O103, and O111 even though these serogroups were present in the rumen. Our results suggest that Tasco effectively reduced pathogenic *E. coli* but had only minimal impacts on rumen fermentation in rams.

**IMPORTANCE** Maintaining product safety and reducing the carbon footprint of production are two sustainability goals of the livestock industry. The objective of this study was to study the impact of Tasco, a product derived from the brown macroalga *Ascophyllum nodosum*, on the rumen microbiome and its function. The inclusion of Tasco altered both rumen and fecal microbiota levels without affecting rumen fermentation. Tasco reduced fecal *Escherichia coli* populations and specifically reduced the prevalence of Shiga toxin-producing *E. coli* O45, O103, O111, and O121 in feces. The findings of this study highlight the application of Tasco as a potential feed additive to reduce pathogen shedding in rams without interfering with ruminal metabolism.

## Abstract (precis)

Maintaining product safety and reducing the carbon footprint of production are two sustainability goals of the livestock industry. The objective of this study was to study the impact of Tasco, a product derived from the brown macroalga *Ascophyllum nodosum*, on the rumen microbiome and its function. The inclusion of Tasco altered both rumen and fecal microbiota levels without affecting rumen fermentation. Tasco reduced fecal *Escherichia coli* populations and specifically reduced the prevalence of Shiga toxin-producing *E. coli* O45, O103, O111, and O121 in feces. The findings of this study highlight the application of Tasco as a potential feed additive to reduce pathogen shedding in rams without interfering with ruminal metabolism.

## INTRODUCTION

Improving the efficiency of rumen microbial fermentation, a process supplying the host with energy in the form of volatile fatty acids (VFAs) and microbial proteins, can enhance the productivity of ruminants. In addition, ruminants are major asymptomatic reservoirs of pathogenic enterohemorrhagic *Escherichia coli* (EHEC) (1). The prevalence of EHEC in cattle can be associated with high-grain feeding (2), which usually results in the production of larger amount of lipopolysaccharide in the rumen (3). The high prevalence of EHEC in ruminants has led to public concern about meat and milk safety (4). Identification of a feed additive that improves animal productivity by enhancing rumen microbial fermentation efficiency and reduces EHEC population size could offer significant value.

A sun-dried seaweed extract (Tasco; Acadian Seaplants Ltd., Dartmouth, NS, Canada) containing a mixture of polysaccharides and oligosaccharides and derived from *Ascophyllum nodosum* has been reported to have a positive effect on the immune function and health of lambs when fed at <1% of their diet dry matter (DM) (5). Moreover, goats fed *A. nodosum* extract at 2% of their diet DM exhibited greater heat tolerance and reduced transport-induced oxidative stress (6). When Tasco was included in beef cattle diets at 2% of the DM, a higher marbling score and a higher meat quality grade were also observed (7). Previous research also has shown that Tasco reduces *E. coli* O157:H7 shedding in steers (8). However, despite all of these studies, the impact of Tasco on the rumen microbiome is unknown.

We hypothesize that Tasco can alter the rumen microbiota, leading to greater fermentation activity while inhibiting the *E. coli* O157 and non-O157 serogroups in the rumen. Such a reduction in ruminal *E. coli* could possibly reduce the fecal shedding of these food-borne pathogens. Therefore, in this study, we evaluated the effects of Tasco on the rumen microbiome, as well as the presence of seven *E. coli* O serogroups, including O157 and six non-O157 *E. coli* serogroups (O26, O45, O103, O111, O121, and O145) in the rumen and feces by using rams as an experimental model.

## RESULTS

The components and chemical composition of the diets used in this study are listed in Table 1. In all of the tables and figures in Results and in the Discussion, the four diets are referred to as follows: Con, control; 1SW, 1% DM-based Tasco; 3SW, 3% DM-based Tasco; 5SW, 5% DM-based Tasco.

**TABLE 1:** Ingredients and chemical compositions of diets

Item	Mean % of diet^a^ DM ± SD (*n* = 4)			
	Con	1SW	3SW	5SW
Ingredient				
Mixed hay^b^	4.00	4.00	4.00	4.00
Barley grain, ground	48.40	47.40	45.40	43.40
Dehydrated alfalfa, ground	35.90	35.90	35.90	35.90
Beet pulp, dehydrated	6.70	6.70	6.70	6.70
Beet molasses	2.40	2.40	2.40	2.40
Tasco-AOS meal		1.00	3.00	5.00
Mineral premix^c^	1.00	1.00	1.00	1.00
Dicalcium phosphate	0.600	0.600	0.600	0.600
Calcium carbonate	0.500	0.500	0.500	0.500
Ammonium chloride	0.475	0.475	0.475	0.475
Vitamins A, D, E^d^	0.025	0.025	0.025	0.025
Chemical composition^e^				
% DM	92.4 ± 0.82	91.9 ± 1.02	91.8 ± 1.04	92.2 ± 0.69
Organic matter	91.3 ± 0.30	91.5 ± 0.34	91.5 ± 0.38	90.9 ± 0.26
CP	15.4 ± 0.80	15.7 ± 0.68	15.7 ± 0.86	15.5 ± 0.91
NDF	26.9 ± 0.42	27.1 ± 0.98	28.3 ± 0.98	28.3 ± 1.21
ADF	16.1 ± 0.91	16.8 ± 0.34	17.5 ± 0.53	18.4 ± 0.69
Ether extract	2.6 ± 0.32	2.6 ± 0.12	2.8 ± 0.74	2.8 ± 0.37

a Con, control; 1SW, 1% DM-based Tasco; 3SW, 3% DM-based Tasco; 5SW, 5% DM-based Tasco.

b Diets, except mixed hay, were pelleted. Hay and pellets were offered at a ratio of 0.04:0.96 (DM basis).

c Contained (all on a DM basis) salt (92.7%), Dynamate (5.0%), zinc sulfate (0.9%), manganese sulfate (0.8%), canola oil (0.4%), selenium premix (0.1%), ethylenediamine dihydroiodide (0.01%), and cobalt carbonate (0.004%).

d Contained (all on a DM basis) vitamin A (9,900,000 IU/kg), vitamin D (990,000 IU/kg), and vitamin E (9,900 IU/kg).

e Values are averages and standard deviations.

### Changes in digestion parameters and rumen microbial fermentation in response to Tasco.

Feed consumption by all rams was monitored throughout the experiment, and no signs of feed sorting were noted. With increasing levels of Tasco, feed intake increased linearly (*P* < 0.01) while the digestibility of DM, organic matter (OM), neutral detergent fiber (NDF), and acid detergent fiber (ADF) was unaffected and crude protein (CP) digestibility decreased linearly (*P* = 0.006) (Table 2). The actual Tasco intake was approximately 0, 18, 57, and 100 g/day for the Con, 1SW, 3SW, and 5SW diets, respectively. In addition, body weight gain (BWG) increased numerically with increasing levels of Tasco. The total VFA concentrations were similar, whereas the molar proportion of most of the individual VFAs differed among the four diets; acetate was increased, while propionate, butyrate, isovalerate, and isobutyrate were decreased with increasing levels of Tasco. Rumen NH_3_-N concentrations differed among the diets, but no linear or quadratic effects were observed. Rumen pH was not affected by Tasco.

**TABLE 2:** Different feed intake by and fermentation parameters of groups of eight rams fed Con, 1SW, 3SW, and 5SW diets^a^

Parameter	Con	1SW	3SW	5SW	SEM	*P* value		
						Diets	Linear	Quadratic
Intake (g/day) of:								
DM	1,841	1,759	1,893	2,013	119	<0.001	<0.001	0.075
OM	1,688	1,610	1,733	1,830	109	<0.001	<0.001	0.114
CP	286	276	299	310	22	0.002	0.001	0.400
NDF	493	478	536	569	29	<0.001	<0.001	0.312
ADF	294	295	332	371	22	<0.001	<0.001	0.222
% Digestibility of:								
DM	76.1	75.0	73.9	71.3	1.67	0.105		
OM	78.2	77.5	76.2	73.9	1.4	0.100		
CP	70.9	69.9	67.6	64.0	2.0	0.041	0.006	0.693
NDF	51.7	50.6	52.3	46.5	3.5	0.469		
ADF	40.6	42.3	42.0	36.9	3.9	0.530		
BWG (kg/day)	0.21	0.23	0.25	0.26	0.02	0.789		
Fermentation								
Total VFAs, mM	142	155	154	144	9	0.230		
Acetate, mol/100 mol	50.2	56.7	55.1	57.8	1.5	<0.001	<0.001	0.088
Propionate, mol/100 mol	27.9	21.5	27.1	22.8	2.2	<0.001	0.046	0.968
Butyrate, mol/100 mol	18.3	18.0	14.5	15.9	2.0	<0.001	<0.001	0.029
Valerate, mol/100 mol	2.00	2.03	2.03	1.87	0.34	0.757		
Isovalerate, mol/100 mol	0.59	0.62	0.36	0.48	0.08	<0.001	<0.001	0.003
Isobutyrate, mol/100 mol	0.64	0.62	0.40	0.53	0.09	<0.001	<0.001	<0.001
Acetate/propionate ratio	1.94	2.83	2.31	2.72	0.22	<0.001	0.002	0.178
NH_3_-N concn, mM	6.5	12.5	8.5	10.6	2.8	<0.001	0.159	0.238
pH	5.93	5.86	5.89	6.02	0.06	0.804		

a Con, control; 1SW, 1% DM-based Tasco; 3SW, 3% DM-based Tasco; 5SW, 5% DM-based Tasco.

### Tasco reduced bacterial and archaeal populations and increased protozoal populations in the rumen.

As shown in Table 3, feeding rams Tasco linearly reduced (*P* < 0.001) their total bacterial and archaeal populations, as estimated by the total copy number of 16S rRNA genes. Contradictorily, the protozoal population (estimated by the number of cells per milliliter of rumen fluid) increased linearly with Tasco (*P* < 0.001), with protozoa approximately 4-fold more abundant in Tasco-fed than in Con-fed rams.

**TABLE 3:** Total bacterial and archaeal populations in the rumens of rams fed increasing levels of *A. nodosum* extract

Population	Con^a^	1SW	3SW	5SW	*P* value	
					Linear	Quadratic
Bacteria	11.63 ± 0.11^b^	10.43 ± 0.20	10.64 ± 0.20	10.49 ± 0.17	<0.001	0.006
Archaea	8.70 ± 0.05^b^	8.54 ± 0.07	8.25 ± 0.09	8.12 ± 0.07	<0.001	0.231
Protozoa	3.40 ± 0.66^c^	4.53 ± 0.56	4.77 ± 0.56	5.14 ± 0.63	<0.001	0.060

a Con, control; 1SW, 1% DM-based Tasco; 3SW, 3% DM-based Tasco; 5SW, 5% DM-based Tasco.

b Data are presented as a log_10_ number of 16S rRNA gene copies/g conversion.

c Data are presented as a log_10_ number of cells/ml of rumen fluid conversion.

### Effect of Tasco on rumen bacterial, archaeal, and protozoal communities.

**FIG 1:**
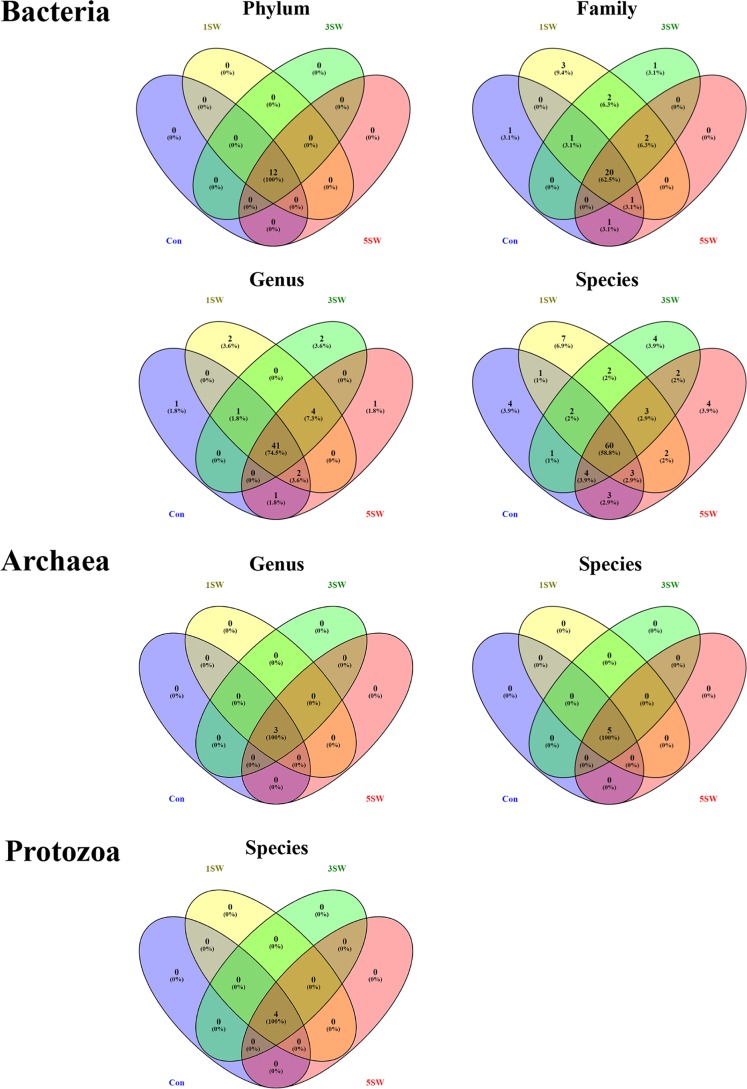
Distribution of microbial phylotypes among differed seaweed levels. Venn diagrams were constructed for all of the microbial phylotypes analyzed, including bacterial phylotypes at the phylum, family, genus, and species levels and archaeal phylotypes at the genus and species levels. Among the phylotypes analyzed, those of 12 bacterial phyla, 20 bacterial families, 41 bacterial genera, and 60 bacterial species; 5 archaeal species; and 5 protozoal species were present in all four diets and were considered the “core microbiota.”

A total of 688,954 bacterial reads were assigned to 45,031 unique operational taxonomic units (OTUs) that belonged to 25 phyla, 184 families, 294 genera, and 310 species. As for the archaeal community, 85,111 archaeal reads passed the quality filter and were assigned to 334 unique OTUs belonged to 4 genera and 7 species. All of the 1,257,183 protozoal reads were assigned to 50 unique OTUs belonging to 7 species. The overall Chao1 index, Good’s coverage, and Simpson indices did not differ between the Con and treatment groups of bacteria, archaea, or protozoa (data not shown). Of the phylotypes identified, only those with a relative abundance of >0.01% and identified in at least four samples of at least one treatment group were considered “present” and maintained for downstream analyses. After being filtered against these criteria, bacterial phylotypes belonging to 12 phyla, 32 families, 55 genera, and 102 species; archaeal phylotypes belonging to 3 genera and 5 species; and 4 protozoal species were subjected to all downstream statistical analyses. The distributions of these identified phylotypes are plotted in Fig. 1. The bacterial phylotypes of 12 phyla, 20 families, 41 genera, and 60 species; the archaeal phylotypes of 3 genera and 5 species; and the protozoal phylotypes of 4 species were present in all of the samples.

**FIG 2:**
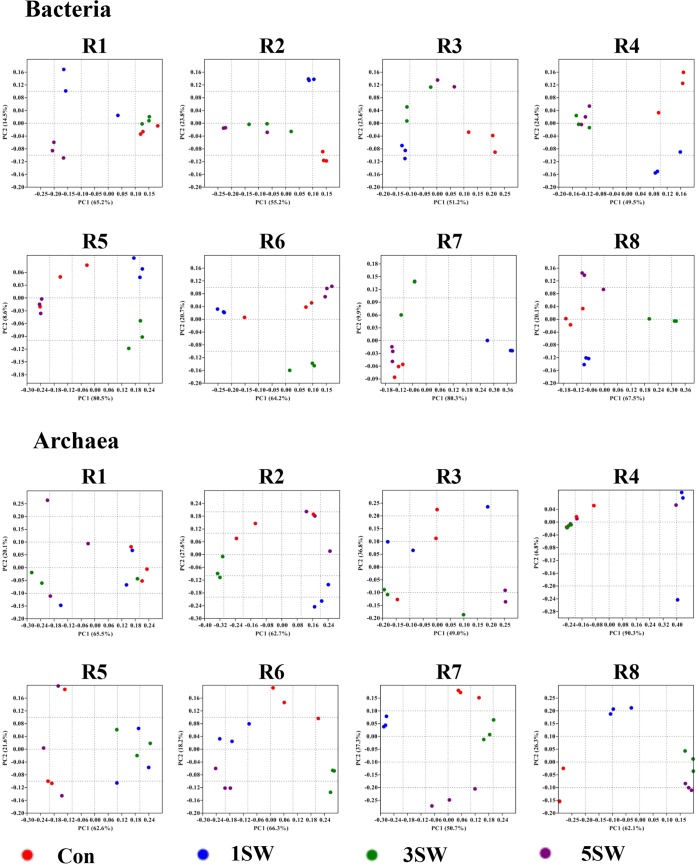
PCoA clustering for the bacterial and archaeal profiles at the species level. R1 to R8 are ram identification numbers. Tasco-based clusters were observed in most of the animals.

The bacterial profiles and archaeal profiles were similar between the baseline samples (before treatment) and the Con samples for each ram, respectively (all *P* > 0.1, Table 4). Principal-coordinate analysis (PCoA) showed that both bacterial and archaeal profiles clustered according to Tasco levels (Fig. 2), and diet-wise analysis of similarity (ANOSIM) supported this clustering, where most of the *P* values indicated a trend or significance (Table 4).

**TABLE 4:** Diet-wise comparison of the overall microbial profiles of individual rams indicates Tasco level-driven clustering

Population and ram	ANOSIM R (*P* value)^a^							
	Base vs Con	Con vs 1SW	Con vs 3SW	Con vs 5SW	1SW vs 3SW	1SW vs 5SW	3SW vs 5SW	Overall
Bacteria								
R1	1.000 (0.250)	0.889 (0.101)	0.963 (0.100)	1.000 (0.106)	0.815 (0.100)	0.815 (0.101)	1.000 (0.101)	0.923 (0.010)
R2	1.000 (0.254)	1.000 (0.106)	0.630 (0.101)	1.000 (0.096)	0.704 (0.100)	1.000 (0.098)	0.259 (0.307)	0.818 (0.010)
R3	1.000 (0.256)	1.000 (0.098)	1.000 (0.099)	1.000 (0.101)	0.815 (0.101)	1.000 (0.097)	0.500 (0.198)	0.893 (0.010)
R4	1.000 (0.249)	1.000 (0.103)	1.000 (0.101)	1.000 (0.101)	1.000 (0.100)	1.000 (0.097)	1.000 (0.103)	1.000 (0.010)
R5	1.000 (0.252)	1.000 (0.100)	1.000 (0.097)	0.444 (0.099)	1.000 (0.106)	1.000 (0.102)	1.000 (0.105)	0.895 (0.010)
R6	0.111 (0.746)	0.667 (0.103)	0.519 (0.096)	0.481 (0.100)	1.000 (0.098)	1.000 (0.100)	1.000 (0.102)	0.787 (0.010)
R7	1.000 (0.249)	1.000 (0.096)	1.000 (0.104)	0.926 (0.096)	1.000 (0.099)	1.000 (0.099)	0.852 (0.101)	0.877 (0.010)
R8	1.000 (0.253)	1.000 (0.094)	1.000 (0.096)	0.926 (0.100)	1.000 (0.100)	1.000 (0.096)	1.000 (0.096)	0.951 (0.010)
Archaea								
R1	1.000 (0.251)	0.296 (0.206)	0.481 (0.098)	0.481 (0.100)	0.815 (0.100)	0.222 (0.196)	0.148 (0.299)	0.302 (0.034)
R2	1.000 (0.252)	0.963 (0.100)	0.666 (0.100)	0.259 (0.295)	1.000 (0.100)	1.000 (0.103)	1.000 (0.103)	0.833 (<0.001)
R3	−0.333 (0.748)	−0.037 (0.500)	0.296 (0.199)	0.296 (0.199)	0.296 (0.100)	1.000 (0.104)	0.750 (0.099)	0.436 (0.105)
R4	0.111 (0.496)	1.000 (0.100)	0.407 (0.094)	0.259 (0.204)	1.000 (0.096)	0.630 (0.100)	0.556 (0.102)	0.583 (<0.001)
R5	0.111 (0.501)	0.704 (0.097)	0.852 (0.098)	−0.333 (1.000)	0.370 (0.097)	0.852 (0.102)	0.778 (0.097)	0.540 (0.003)
R6	1.000 (0.250)	0.667 (0.103)	0.519 (0.096)	0.481 (0.100)	1.000 (0.098)	1.000 (0.100)	1.000 (0.102)	0.991 (<0.001)
R7	1.000 (0.249)	1.000 (0.097)	1.000 (0.102)	1.000 (0.098)	1.000 (0.098)	1.000 (0.102)	0.889 (0.104)	0.957 (<0.001)
R8	0.556 (0.253)	1.000 (0.100)	1.000 (0.101)	1.000 (0.106)	1.000 (0.097)	1.000 (0.095)	1.000 (0.096)	0.988 (<0.001)

a Base, baseline; Con, control; 1SW, 1% DM-based Tasco; 3SW, 3% DM-based Tasco; 5SW, 5% DM-based Tasco. ANOSIM R, dissimilarity index.

### Effect of Tasco on rumen bacterial, archaeal, and protozoal taxa.

The relative abundances of the bacterial phylotypes (phylum, family, genus, and species levels) and archaeal phylotypes (genus and species levels) at different Tasco levels were then compared. At the phylum level, the most abundant phyla were *Bacteroidetes*, *Firmicutes*, and *Proteobacteria*, which were present at similar levels in all four diet groups (see Table S1 in the supplemental material). The proportions of *Actinobacteria* (*P* = 0.006) and *Tenericutes* (*P* = 0.003) were linearly reduced by Tasco, while a quadratic effect of the Tasco level was detected for the phylum TM7 (*P* = 0.004) (Table S1). A dosage effect of Tasco was observed in only four bacterial families (Table S2); a linear effect was observed in *Coriobacteriaceae* (linear *P* = 0.004), an undefined family of class TM7-1 (linear *P* = 0.041), and *Paenibacillaceae* (linear *P* = 0.007), whereas a quadratic effect was observed in *Veillonellaceae* (quadratic *P* = 0.023).

10.1128/mSphere.00017-18.5 TABLE S1  *Actinobacteria*, phylum TM7, and *Tenericutes* were linearly reduced in rams fed the 1SW, 3SW, and 5SW diets compared with those fed the Con diet (*n* = 8 per diet). Download TABLE S1, PDF file, 0.1 MB. Copyright © 2018 Zhou et al. 2018 Zhou et al. This content is distributed under the terms of the Creative Commons Attribution 4.0 International license.

10.1128/mSphere.00017-18.6 TABLE S2  Members of the family *Coriobacteriaceae* were reduced, while those of the families *Paenibacillaceae* and *Veillonellaceae* were increased, in rams fed the 1SW, 3SW, and 5SW diets compared with those fed the Con diet (*n* = 8 per diet). Download TABLE S2, PDF file, 0.1 MB. Copyright © 2018 Zhou et al. 2018 Zhou et al. This content is distributed under the terms of the Creative Commons Attribution 4.0 International license.

At the genus level, the relative abundance of most of the bacterial phylotypes remained stable at different Tasco levels. As shown in Table S3, a linear effect was seen in *Roseburia* (linear *P* = 0.020), *Syntrophococcus* (linear *P* = 0.010), and an undefined genus of the class TM7-1 (linear *P* = 0.041), while a quadratic effect was seen in an undefined genus of the family *Paenibacillaceae* (quadratic *P* = 0.023), an undefined genus of the order RF39 (quadratic *P* = 0.018), and *Syntrophococcus* (quadratic *P* = 0.011). In addition, the relative abundance of all three archaeal genera was not affected by the level of Tasco.

10.1128/mSphere.00017-18.7 TABLE S3  *Roseburia*, *Syntrophococcus*, and an undefined genus of the class TM7-1 were linearly reduced, while members of the families *Paenibacillaceae* and *Veillonellaceae* were increased in rams fed the 1SW, 3SW, and 5SW diets compared with those fed the Con diet (*n* = 8 per diet). Download TABLE S3, PDF file, 0.1 MB. Copyright © 2018 Zhou et al. 2018 Zhou et al. This content is distributed under the terms of the Creative Commons Attribution 4.0 International license.

**FIG 3:**
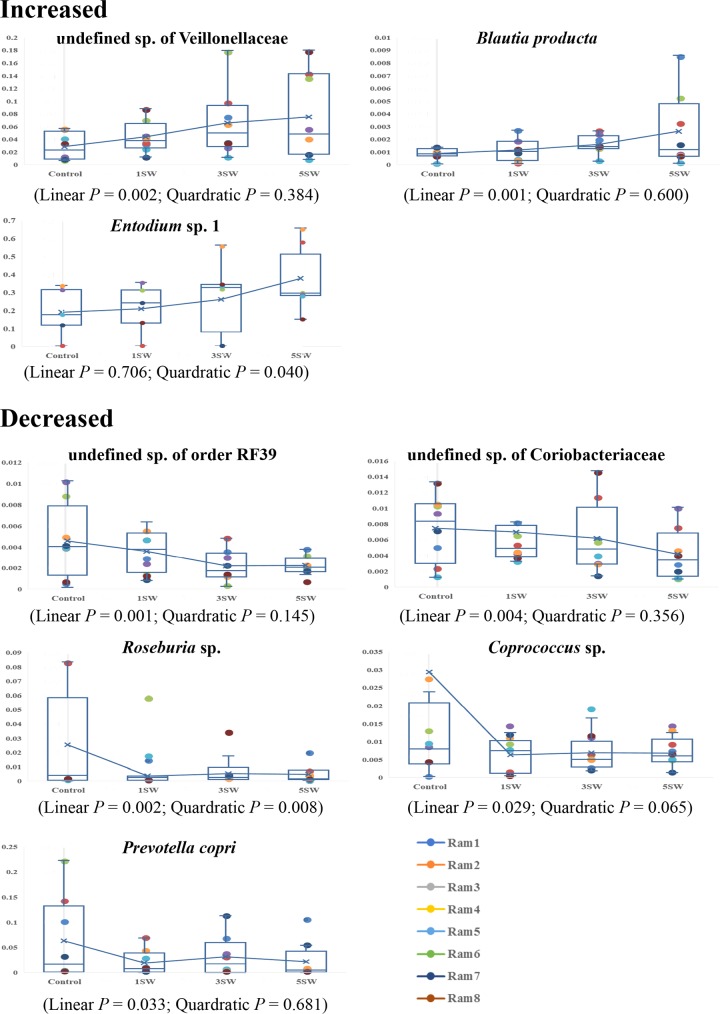
Tasco-affected microbial species. Mean values for each diet and data collected from individual rams were plotted. Most of the changing trends for these eight species observed in each animal were similar to those found for the mean values. On the *y* axis is the proportion of the community accounted for by the species.

At the species level, only nine species were found to be affected by the Tasco dose; the relative abundances of an undefined species of the family *Veillonellaceae* (linear *P* = 0.002), *Blautia producta* (linear *P* = 0.001), and *Entodinium* species 1 (quadratic *P* = 0.040) were increased by increasing levels of Tasco, while the relative abundances of an undefined species of the order RF39 (linear *P* = 0.001), an undefined species of the family *Coriobacteriaceae* (linear *P* = 0.004), *Roseburia* sp. (linear *P* = 0.002; quadratic *P* = 0.008), *Coprococcus* sp. (linear *P* = 0.029), and *Prevotella copri* (linear *P* = 0.033) were decreased (Fig. 3). The relative abundances of the remaining 101 bacterial species, 5 archaeal species, and 3 protozoal species were similar among all of the diets (Table S4).

10.1128/mSphere.00017-18.8 TABLE S4  Similar relative abundances of 101 out of 108 bacterial species, 5 archaeal species, and 3 protozoal species in rams fed the 1SW, 3SW, and 5SW diets and those fed the Con diet (*n* = 8 per diet). Download TABLE S4, PDF file, 0.1 MB. Copyright © 2018 Zhou et al. 2018 Zhou et al. This content is distributed under the terms of the Creative Commons Attribution 4.0 International license.

### Functional potential of the rumen microbiota and effect of Tasco on predicted functional pathways.

**FIG 4:**
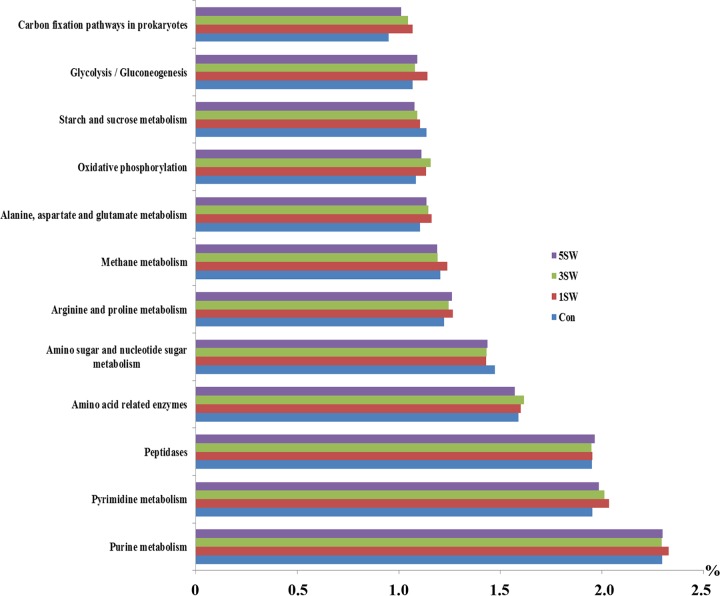
Relative abundances of the major microbial KEGG metabolic pathways. The values on the *x* axis indicate the proportions of the functions listed.

In total, 328 KEGG pathways were predicted from the entire data set by PICRUSt (9), with nearest sequenced taxon index values ranging between 0.085 and 0.248 (0.166 ± 0.004) among all samples. Of the 328 predicted pathways, 84 were metabolism associated, with a relative abundance of >0.1%. The metabolic pathways with an average proportion of >1.0% were defined as major, whereas those with an average proportion between 0.1% and 1.0% were defined as minor. On the basis of this definition, 12 metabolic pathways were considered major, including those related to protein, carbohydrate, and methane metabolism (Fig. 4). Among these 12 major metabolic pathways, the Tasco level had a quadratic effect (*P* = 0.002) on only 1, “carbon fixation pathways in prokaryotes” (Table 5, underlined). In addition, 16 minor pathways were affected by Tasco; of these, 13 were more abundant and 3 were less abundant (Table 5) in the rumens of Tasco-fed rams.

**TABLE 5:** Fourteen metabolic pathways were increased and three were reduced with increasing levels of *A. nodosum* (Tasco) in the diet^a^

Trend between Con and treatment and predicted function	Con	1SW	3SW	5SW	*P* value	
					Linear	Quadratic
Increased						
Carbon fixation pathways in prokaryotes^b^	0.95 ± 0.01	1.07 ± 0.01	1.05 ± 0.01	1.01 ± 0.01	0.291	0.002
Histidine metabolism	0.60 ± 0.01	0.67 ± 0.01	0.66 ± 0.01	0.64 ± 0.02	0.223	<0.001
Phenylalanine metabolism	0.18 ± 0.00	0.20 ± 0.00	0.20 ± 0.00	0.20 ± 0.01	0.055	0.004
Tyrosine metabolism	0.31 ± 0.01	0.36 ± 0.01	0.35 ± 0.01	0.33 ± 0.01	0.735	0.003
Tropane, piperidine and pyridine alkaloid biosynthesis	0.11 ± 0.00	0.13 ± 0.00	0.13 ± 0.00	0.12 ± 0.01	0.214	0.008
Butanoate metabolism	0.56 ± 0.01	0.60 ± 0.01	0.58 ± 0.01	0.60 ± 0.01	0.027	0.491
Fatty acid biosynthesis	0.44 ± 0.02	0.50 ± 0.01	0.48 ± 0.02	0.47 ± 0.02	0.530	0.041
Fatty acid metabolism	0.18 ± 0.01	0.20 ± 0.00	0.19 ± 0.00	0.20 ± 0.00	0.005	0.326
Nicotinate and nicotinamide metabolism	0.47 ± 0.01	0.51 ± 0.01	0.79 ± 0.01	0.48 ± 0.02	0.932	0.036
Pantothenate and CoA^c^ biosynthesis	0.65 ± 0.01	0.68 ± 0.01	0.69 ± 0.01	0.67 ± 0.02	0.244	0.028
Vitamin B_6_ metabolism	0.22 ± 0.01	0.24 ± 0.01	0.24 ± 0.00	0.24 ± 0.01	0.382	0.040
Prenyl transferases	0.32 ± 0.01	0.36 ± 0.01	0.36 ± 0.01	0.34 ± 0.02	0.410	0.040
Naphthalene degradation	0.12 ± 0.01	0.17 ± 0.01	0.15 ± 0.01	0.15 ± 0.01	0.189	0.047
Fructose and mannose metabolism	0.90 ± 0.02	0.98 ± 0.02	0.94 ± 0.01	0.91 ± 0.02	0.781	0.016
Decreased						
Glycerophospholipid metabolism	0.52 ± 0.01	0.50 ± 0.01	0.50 ± 0.00	0.49 ± 0.01	0.026	0.811
Porphyrin and chlorophyll metabolism	0.91 ± 0.04	0.74 ± 0.03	0.81 ± 0.04	0.85 ± 0.03	0.931	0.021
Seleno compound metabolism	0.37 ± 0.01	0.34 ± 0.01	0.34 ± 0.01	0.35 ± 0.00	0.221	0.032

a DM basis; *n* = 8 per diet. Con, control; 1SW, 1% DM-based Tasco; 3SW, 3% DM-based Tasco; 5SW, 5% DM-based Tasco. Data in columns 2 to 5 are average percentages and standard deviations.

b Major metabolic pathway.

c CoA, coenzyme A.

### Correlation among microbial abundance, fermentation parameters, and predicted microbial functions.

Moderate correlations between the relative abundances of archaeal and protozoal species were found. *Methanosphaera* sp. was positively correlated with *Dasytricha* sp. (*r* = 0.513, *P* < 0.001) and an undefined species of “*Methanoplasmatales*” was positively correlated with *Isotricha* species 1 (*r* = 0.590, *P* < 0.001). Bacterial species did not linearly correlate with either archaeal or protozoal species. Rather, positive or negative co-occurrence patterns were identified between bacterial species and archaeal/protozoal species (Fig. S1).

10.1128/mSphere.00017-18.1 FIG S1  Co-occurrence patterns of the microbial phylotypes. Only statistically significant co-occurrences are plotted. Blue indicates that the two phylotypes are very likely to co-occur in one sample, yellow indicates that the two phylotypes are very unlikely to co-occur in one sample, and gray indicates that the co-occurrence patterns of the two phylotypes are random. The value in each box is the *P* value. Download FIG S1, PDF file, 0.2 MB. Copyright © 2018 Zhou et al. 2018 Zhou et al. This content is distributed under the terms of the Creative Commons Attribution 4.0 International license.

Correlations between microbial populations and metabolites were also identified. The total protozoal population tended to be positively correlated with the acetic acid molar portion (*r* = 0.498, *P* < 0.001) and acetate/propionate ratio (*r* = 0.641, *P* < 0.001) and negatively correlated with the propionic acid molar portion (*r* = −0.573, *P* < 0.001). A protozoal *Polyplastron* sp. was positively correlated with the NH_3_-N concentration (*r* = 0.550, *P* < 0.001), and *Isotricha* species 2 was positively correlated with the molar portion of valeric acid (*r* = 0.538, *P* < 0.001). Trends of correlations (0.3 ≤ *r* < 0.5 with *P* < 0.01) were observed between individual microbial species and the molar portions of individual VFAs (Fig. S2). In addition, correlations between individual VFAs and bacterial phyla were also observed (Fig. S3).

10.1128/mSphere.00017-18.2 FIG S2  Correlations among individual VFA concentrations, NH_3_-N concentrations, and the relative abundances of individual bacterial species. Only statistically significant correlations are plotted. Values are correlation coefficients. Green, moderate correlation (0.5 ≤ *r* < 0.7 with *P* < 0.001); yellow, trend of correlation (0.3 < *r* ≤ 0.5 with *P* < 0.01). Download FIG S2, PDF file, 0.1 MB. Copyright © 2018 Zhou et al. 2018 Zhou et al. This content is distributed under the terms of the Creative Commons Attribution 4.0 International license.

10.1128/mSphere.00017-18.3 FIG S3  Correlations among individual VFAs and bacterial phyla. Correlation coefficients are presented in gradient colors, and the *P* values for the correlations are shown. Download FIG S3, PDF file, 0.8 MB. Copyright © 2018 Zhou et al. 2018 Zhou et al. This content is distributed under the terms of the Creative Commons Attribution 4.0 International license.

The relationship between the Tasco-affected microbial phylotypes and predicted microbial metabolic pathways was also explored. As listed in Table 6, moderate correlations were observed between the relative abundance of *Roseburia* sp. and glycerophospholipid metabolism (*r* = 0.588, *P* < 0.001), between the relative abundance of *Entodinium* species 1 and “valine, leucine, and isoleucine biosynthesis,” “pyruvate metabolism,” “fatty acid metabolism,” “chloroalkane and chloroalkene degradation,” and “naphthalene degradation.” The trends of correlations between Tasco-affected microbial species and microbial metabolic pathways are also listed in Table 6. Associations between bacterial phylotypes at the family and genus levels were also observed for selected metabolic pathways (Table S5).

10.1128/mSphere.00017-18.9 TABLE S5  Correlation between the relative abundances of microbial phylotypes at different phylogenic levels and the predicted KEGG metabolic pathways. Download TABLE S5, PDF file, 0.1 MB. Copyright © 2018 Zhou et al. 2018 Zhou et al. This content is distributed under the terms of the Creative Commons Attribution 4.0 International license.

**TABLE 6:** Correlation between the relative abundance and predicted functional abundance of bacterial and archaeal species

Organisms	Function	Correlation coefficient	*P* value
Bacteria			
*Roseburia* sp.	Glycerophospholipid metabolism	0.5878	<0.001
	Porphyrin and chlorophyll metabolism	0.3808	<0.001
	Seleno compound metabolism	0.4166	<0.001
*Coprococcus* sp.	Glycerophospholipid metabolism	0.3973	<0.001
	Porphyrin and chlorophyll metabolism	0.2684	0.009
*Prevotella copri*	Seleno compound metabolism	0.3859	<0.001
*Blautia producta*	Fructose and mannose metabolism	0.2715	0.008
Undefined archaeal “*Methanoplasmatales*” species	Lysine degradation	0.3558	<0.001
	Butanoate metabolism	0.3069	0.003
		
Protozoon *Entodinium* species 1	Arginine and proline metabolism^a^	0.4221	0.005
	Glycolysis/gluconeogenesis^a^	0.4061	0.008
	Valine, leucine, and isoleucine biosynthesis	0.5150	<0.001
	Streptomycin biosynthesis	0.3303	0.033
	Butanoate metabolism	0.5058	<0.001
	Tyrosine metabolism	0.3508	0.023
	Propanoate metabolism	0.3512	0.023
	Pyruvate metabolism	0.5171	<0.001
	Methane metabolism	0.4131	0.007
	Unsaturated fatty acid biosynthesis	0.3845	0.012
	Fatty acid biosynthesis	0.3360	0.030
	Fatty acid metabolism	0.5034	<0.001
	Lipid biosynthesis proteins	0.4409	0.004
	Benzoate degradation	0.3353	0.030
	Chloroalkane and chloroalkene degradation	0.5793	<0.001
	Naphthalene degradation	0.5575	<0.001

a Major microbial KEGG metabolic pathway.

### Effects of Tasco on the total ruminal *E. coli* population and on the presence of ruminal and fecal *E. coli* O serogroups.

Increasing Tasco reduced the total *E. coli* population in the rumens of rams in both linear (*P* < 0.001) and quadratic (*P* = 0.002) manners (Table 7). Overall *E. coli* O serogroups in the rumen and feces responded differentially to Tasco (Fig. S3). Detection of an *E. coli* O serogroup in the rumen did not necessarily result in its detection in feces (Table S6). No significant co-occurrence pattern (either positive nor negative) was observed in any *E. coli* O serogroup in either the rumen or feces.

10.1128/mSphere.00017-18.10 TABLE S6  Co-occurrence of *E. coli* O serotypes in rumen and fecal samples from rams fed different levels of seaweed (DM basis; *n* = 8 per diet). Download TABLE S6, PDF file, 0.1 MB. Copyright © 2018 Zhou et al. 2018 Zhou et al. This content is distributed under the terms of the Creative Commons Attribution 4.0 International license.

**TABLE 7:** Rumen total *E. coli* population decreases with increasing levels of Tasco in the diet^a^

Avg population decrease ± SD				*P* value	
Con	1SW	3SW	5SW	Linear	Quadratic
6.82 ± 0.09	6.27 ± 0.14	5.86 ± 0.12	5.90 ± 0.13	<0.001	0.002

a DM basis; *n* = 8 per diet. Con, control; 1SW, 1% DM-based Tasco; 3SW, 3% DM-based Tasco; 5SW, 5% DM-based Tasco. Data are presented as a log_10_ copies of 16S rRNA gene/g conversion.

Tasco had little effect on the colonization of the seven *E. coli* O serogroups in the rumen (Table 8). For instance, O26 was detected in only one sample from rams fed the Con diet, but it was also detected in the same ram when it was fed the 5SW diet. O157 was detected in six out of eight rams when they were fed the Con diet. Even when rams were fed the 1SW, 3SW, and 5SW diets, at least half of the rumen samples were still O157 positive. Feeding higher levels of Tasco (3SW and 5SW) reduced the prevalence of O45, O103, and O111 in feces. Additionally, O121 was completely depleted from the rumen when 5SW was fed, while it was not detected in feces at either the 3SW or the 5SW level. All four of these O serogroups were detected in fecal samples from rams fed the Con or 1SW diet (Table 8).

**TABLE 8:** Presence/absence of the seven *E. coli* O serogroups examined in rumen and fecal samples with increasing levels of *A. nodosum* (Tasco) in the diet

Diet^a^	O26		O45		O103		O111		O121		O145		O157	
	Ru^b^	Fe^c^	Ru	Fe	Ru	Fe	Ru	Fe	Ru	Fe	Ru	Fe	Ru	Fe
Con	1^d^		3	3	3	1	2	1	1	2	3		6	
1SW		2	3	1	1	1	2	1	2	1	2		7	
3SW			4		5		4		1		1		7	
5SW	1	1	3		3		3				1		4	

a Con, control; 1SW, 1% DM-based Tasco; 3SW, 3% DM-based Tasco; 5SW, 5% DM-based Tasco.

b Ru, rumen.

c Fe, feces.

d Values are the number of samples positive among the eight samples examined per diet.

### Indication of competition between *E. coli* O serogroups and major microbial phylotypes in the rumen.

Co-occurrence analysis was also performed to determine whether the seven *E. coli* O serogroups displayed patterns of co-occurrence with bacteria within the rumen. As shown in Table 9, O45, O121, and O157 displayed patterns of co-occurrence with bacterial phylotypes; O45 was unlikely to co-occur with *Succiniclasticum* sp.; O121 was unlikely to co-occur with *Shuttleworthia* or *Megasphaera* sp., and O157 was very likely to co-occur with *Dialister* sp.

**TABLE 9:** Co-occurrence of *E. coli* O serogroups and bacterial species in the rumen

*E. coli* serogroup	Bacterial species	Co-occurrence	*P* value
O45	*Succiniclasticum* sp	Negative	0.044
O121	*Shuttleworthia* sp	Negative	0.042
O121	*Megasphaera* sp	Negative	0.025
O157	*Dialister* sp	Positive	0.039

## DISCUSSION

Positive effects, including enhancement of host immunity (9–11), protection of animals from heat or transport-induced stress (5, 6), and reduction of *E. coli* O157:H7 shedding in feces (8), have been reported in ruminants fed *A. nodosum* meal. However, the mechanisms behind these responses remain undefined. This study was therefore conducted to reveal how different levels of Tasco may change host parameters, including intake, digestion, rumen fermentation, rumen microbial populations, and predicted functions.

*A. nodosum* meal was not very palatable for calves when mixed in a starter diet (12), but in this study, no adverse effects on the eating behavior of rams were observed when it was mixed into a complete feed. Rather, there was a linear increase in DM intake (DMI) with increasing levels of Tasco (Table 2), suggesting that it enhanced feed consumption. When assessed in *in vitro* rumen batch cultures, *A. nodosum* did not affect total VFA production, pH, or individual VFAs (13), which was in disagreement with the finding of this study that the molar portions of individual VFAs differed at different Tasco levels (Table 2). Therefore, characterizing the ecology of the rumen microbiome may provide some explanation of the mechanism whereby Tasco alters rumen fermentation profiles *in vivo*.

With comparable total VFA production, the bacterial populations were expected to be similar at the four Tasco levels. Surprisingly, feeding Tasco significantly reduced the bacterial populations (Table 3), contradicting previous *in vitro* studies, where inclusion of either *A. nodosum* or pure phlorotannin (PT) extracts of Tasco resulted bacterial population sizes similar to (8) or even larger than (13) those of control samples. As *A. nodosum* contains fucoidans, laminarin, and PTs, which possess antimicrobial activities (14), the whole Tasco product employed in this study may have retained antimicrobial activities that reduced the bacterial population. Meanwhile, Tasco may have prebiotic effects that enhance the fermentation efficiencies of the remaining bacteria, thus leading to the consistent VFA production seen among diets.

The reduced archaeal population is in accordance with previous *in vitro* studies suggesting that the presence of PTs may be the key factor that depresses archaeal communities. The larger protozoal population observed with Tasco may offer an explanation as to why VFA production remained similar among diets despite the fact that Tasco reduced total bacterial populations. Ciliate protozoa have significant fibrolytic activity (13) and predate bacteria (15); as a result, the significant increase in protozoal numbers with Tasco may have compensated for any bacterium-mediated decline in fiber digestion owing to the reduced bacterial population. However, contradictory results were seen *in vitro*, where the protozoal population was reduced by *A. nodosum* (16). The form of *A. nodosum* (freeze-dried and powdered whole *A. nodosum* versus Tasco) and the experimental system (*in vitro* versus *in vivo*) were different in the two studies, suggesting the necessity of employing an *in vivo* system in future experiments to assess the functions/impacts of Tasco on ruminants.

While the overall VFA production was maintained, the increased protozoal population and the reduced bacterial populations could have reduced the efficiency of CP digestion in the rumen and ultimately resulted in the reduction in CP digestibility (Table 2). The reduced CP digestibility seen may also due to the PTs within Tasco, since it has been previously reported that a tannin-containing supplement tended to reduce the CP digestibility in meat goats (17). We speculated that greater CP intake as a result of greater DMI (Table 2) has led to comparable levels CP-derived nutrients available to the host, which are comparable among the Tasco levels. It should be noted, though, that there was a 10-fold difference in bacterial population size between the Con and Tasco groups, whereas CP digestibility was only slightly reduced (<10%) in Tasco-fed rams. The remaining bacterial communities might function with the promoted protozoal communities through certain mechanisms to maintain the CP digestion capacity within the rumen, but further validation of this hypothesis is required. As the BWG numerically increased along with the increasing levels of Tasco (Table 2) and no signs of unhealthiness were observed in any rams, the reduced CP digestibility caused by Tasco was therefore not considered to have a negative impact on the rams.

The ruminal NH_3_-N concentration can be used as a rough indicator of the efficiency of conversion of dietary N to microbial N (18). The reduced bacterial population size (Table 3) and greater protozoal population size (Table 3) in Tasco-fed rams suggest that protozoal predation of bacteria may have been heightened in the rumens of rams fed Tasco, a possibility that is supported by the higher NH_3_-N concentration in Tasco-fed rams than in Con-fed rams (Table 2). However, although the bacterial population in Con-fed rams was more than 10 times that in Tasco-fed rams, the ruminal NH_3_-N concentration of the Tasco-fed rams was only 30 to 90% greater than that of controls yet within the normal range observed in ovine studies (19–21). Therefore, the higher ruminal NH_3_-N concentration observed in Tasco-fed rams was not considered to have significant adverse environmental or economic effects.

The rumen microbiome was altered when rams received different levels of Tasco, with bacterial phylotypes differing among diets (Fig. 1) and both bacterial and archaeal profiles clustering on the basis of the level of Tasco in the diet for most rams (Fig. 2). This is in agreement with previous *in vitro* experiments where bacterial populations tended to cluster when *A. nodosum* was included as the substrate (16). Downstream analyses showed that Tasco affected the relative abundance of microbial phylotypes at different phylogenic levels (Tables S1 to S3).

In an *in vitro* study where individual bacterial species were inoculated into rumen fluid, the presence of noncellulolytic bacteria such as *Selenomonas ruminantium*, *Ruminobacter amylophilus*, and *Prevotella bryantii* was increased by pure PT extracts from Tasco (13). However, none of these species were altered in the rumens of rams fed a mixed diet containing Tasco. Rather, only two noncellulolytic bacteria and one protozoan (*Entodinium* species 1) were increased, while one cellulolytic bacterium (*Prevotella copri*) and four noncellulolytic bacteria were reduced by Tasco (Fig. 3). Such variation suggests that Tasco, when fed in its original form, may have more complex bioactivities than those solely associated with PT.

The correlations between microbial relative abundance and VFA molar portions identified partly explain how individual VFAs were altered by Tasco. For example, the lower butyrate level observed with Tasco (Table 2) may be explained by the lower relative abundance of the butyrate-producing bacteria *Roseburia* sp. (22) and *Coprococcus* sp. (23) (Fig. 3), as supported by the correlation between *Coprococcus* sp. and butyric acid (Fig. S2). The higher acetate level in Tasco-fed rams (Table 2) may also be attributed to increasing numbers of *Entodinium* species 1 (Fig. 3) and the positive correlation between *Entodinium* species 1 and acetate (*r* = 0.4288, *P* < 0.001, Fig. S2). The negative correlation between the relative abundance of *Firmicutes* and the molar portion of propionate (*r* = −0.4419, *P* < 0.001, Fig. S2) and the numerically but not statistically significantly greater abundance of *Firmicutes* (Table S1) with Tasco (44% in Con versus 51% in Tasco, Table 3) may also have led to the reduced propionate production seen in rams fed Tasco (28 mol/100 mol with Con versus ~23 mol/100 mol with Tasco, Table 2).

With Tasco, the altered predicted metabolic pathways suggest that it may promote microbial fermentation by promoting the upregulation of metabolic pathways. For instance, *A. nodosum* was reported to have a much higher fatty acid concentration (44,670 μg of fatty acids/g of DM) than other macroalgal species (7,262 to 37,413 μg of fatty acids/g of DM) (24). The enriched “fatty acid biosynthesis” and “fatty acid metabolism” pathways observed in seaweed-fed rams (Table 5) indicate that although the overall fatty acid level was low across diets, the rumen microbiome was predicted to have a greater potential to metabolize fatty acid when Tasco was included in the diet. The higher “butanoate metabolism” level of Tasco-fed rams suggests that the microbiome may be more active in metabolizing butyrate, a factor that could have contributed to the reduced butyrate concentration seen in the rumen (Table 2). The more abundant “pantothenate and CoA biosynthesis” may reflect a more active microbiome that requires more energy for these bioactivities. In one of our studies (25), the microbial community was found to be less diverse and less abundant in steers that exhibited greater feed efficiency than their counterparts. We speculated that while Tasco reduced the bacterial population and increased the protozoal population, this shift did not significantly alter rumen fermentation, with the possible exception of N metabolism.

Another key finding of this study is that Tasco reduced the total population of *E. coli* in the rumen, suggesting that changes in the rumen environment in response to ingestion of Tasco are not favorable for its growth. To cover a broader range of Shiga toxin-producing *E. coli* (STEC) strains, the six non-O157 serogroups that were declared the “Big Six” non-O157 STEC strains by the USDA (26) and considered food-borne pathogens were examined together with *E. coli* O157:H7. Owing to the limited sample availability, a multiplex PCR targeting all *E. coli* O serogroups simultaneously, rather than quantitative PCR (qPCR) assays of each individual O serogroup, was used to determine the presence or absence of each O serogroup in rumen and fecal samples. Although some *E. coli* O serogroups have been reported to prevail and/or outcompete opponent strains under certain conditions (27), overall, the total *E. coli* population was reduced 10-fold when Tasco was included in the diet (Table 6). This suggests that this additive causes a broad-spectrum inhibition of *E. coli* within the ruminant digestive tract. Indeed, although the absolute population was not measured, except for O26, the occurrence of the other six O serogroups in fecal samples was reduced by Tasco. For example, although O157 was present in most of the rumen samples regardless of the level of Tasco, none of the fecal samples were O157 positive (Table 7). At this point, it is not known if this reflects outcompetition of O157:H7 by other O serogroups or if it is due to a direct toxic effect of Tasco on O157. In addition, there was no association between the presence of O serogroups in the rumen and their occurrence in feces (Table S4). While the non-O157 serogroups were still positive in the rumen of some rams receiving Tasco, the reduction of generic *E. coli* populations does suggest that it likely reduced the shedding of these undesirable serogroups. *E. coli* may also compete with other bacterial species for metabolic substrates within the rumen, as indicated by the negative cohabitation of O45 and *Succiniclasticum* sp. and of O121 and *Shuttleworthia* sp. and *Megasphaera* sp. (Fig. S2). Promotion of these bacteria may be another way to reduce these corresponding *E. coli* O serogroups.

Reduction of the agricultural carbon footprint has been listed as one of the main targets in building a sustainable agriculture system (28). Increasing levels of Tasco linearly reduced the total methanogens by approximately 30 to 75% (Table 3), a finding comparable to that observed *in vitro* (13) with isolated PT from Tasco. Moreover, a negative correlation was found between *Entodinium* sp. and host CH_4_ and gross energy intake-adjusted CH_4_ in beef cattle (29), so the increase in *Entodinium* sp. caused by Tasco in this study (Fig. 3) may also reflect reduced CH_4_ emission from rams. Recently, it has also been reported that feeding beef cattle seaweed reduced on-farm CH_4_ emission (R. Kinley et al., unpublished data). However, while the greater molar portion of acetate and smaller molar portion of propionate observed in this study (Table 2) were claimed not to be appropriate indicators of daily enteric CH_4_ emissions by sheep (30), studies using emission chambers are therefore needed to assess the impact of Tasco on enteric CH_4_ emissions by rams.

One of the limitations of this study was the lack of animal product measurement. Studying whether Tasco can have a long-term impact on animal production traits such as carcass weight, meat quality, and milk production (for dairy species) may further support our speculation that Tasco has no adverse effect on animal growth. However, the rams used in this study were subjected to other studies after this one, and we were unable to track the animal production data and link them with the Tasco feeding treatment in this study.

In conclusion, feeding Tasco to rams decreased the abundance of rumen bacteria and archaea, increased the abundance of rumen protozoa, and modulated the rumen microbiota by affecting the relative abundance of seven bacterial species and one protozoal species. Different Tasco levels did not affect the BWG of the rams or their total rumen VFA production, but the molar portions of individual VFAs were changed. Ruminal NH_3_-N levels were increased, and the digestibility of CP was decreased. The reason for the alteration of the fermentation parameters may be the fact that with Tasco, the rumen microbiota became more efficient at producing VFAs and less efficient at metabolizing CP. Since no adverse effects on the growth traits of the rams were observed, Tasco was not considered to have any detrimental effects on them. One significant beneficial effect of Tasco feeding was that it reduced the total *E. coli* population in the rumen and reduced the prevalence of O45, O103, O111, and O121 in feces. Our results suggest that Tasco can be used as an effective feed additive to control food-borne pathogens, although it did not enhance rumen microbial fermentation.

## MATERIALS AND METHODS

### Animal experiment and sampling.

The experiment was conducted at the Agriculture and Agri-Food Canada Research Centre in Lethbridge, Alberta, Canada. The experimental protocol was reviewed and approved by the Institutional Animal Care and Use Committee (Ottawa, Ontario, Canada; http://www.ccac.ca/Documents/Standards/Guidelines/Farm_Animals.pdf) and was conducted in accordance with the guidelines of the Canadian Council on Animal Care (31).

Eight Canadian Arcott rams (63.3 ± 4.1 kg) with cannulated rumens were randomly allocated into two groups (four per group) in a four-by-four Latin square design consisting of four 21-day periods and four dietary treatments. Rams were fed *ad libitum* (2% refusal; as-fed basis) once daily at 0800 h during the entire experiment. In each period, rams were adapted to their respective diets for a period of 17 days. After adaptation, total urine and feces collections were carried out between days 18 and 21. Whole rumen contents were collected through a rumen cannula, and fecal samples were collected at 0, 6, and 12 h after feeding on days 19 and 21. The samples collected on day 19 were used for *E. coli* O serogroup screening and bacterial/archaeal profiling, whereas the samples collected on day 21 were filtered through cheesecloth and analyzed for VFAs and NH_3_-N.

Diets were formulated to meet or exceed the nutrient requirements of growing rams (32), and the dietary components used are listed in Table 2. There was no indication of diet sorting as a result of Tasco inclusion in the four diets.

### Rumen digestion, fermentation parameters, and rumen pH measurements.

Animal feed intake and body weight were recorded during each experimental period. Chemical analysis of the major dietary ingredients, the digestibility of the diets, and the rumen fermentation parameters were assessed by using procedures similar to those used by Hünerberg et al. (33). Briefly, diets and ingredients were collected once per week and dried at 55°C for 48 h to analyze DM. Orts were sampled daily and pooled by animals at the end of each experimental period. All samples were frozen at −20°C prior to chemical composition analysis. The apparent total-tract digestibility of each individual ram was assessed by analyzing the DM of the subsample of the daily fecal samples collected between days 18 and 21.

The rumen content samples collected on day 21 at 0, 6, and 12 h after feeding were filtered through two layers of cheesecloth (pore size, 355 μm; B. & S. H. Thompson, Ville-Mont-Royal, Quebec, Canada). A 5-ml volume of filtrate from each filtered sample was mixed with 1 ml of 25% (wt/vol) HPO_3_ to measure VFA, and 5 ml of filtrate was mixed with 1 ml of 1% (wt/vol) H_2_SO_4_ to measure NH_3_-N. Rumen pH was measured with the LRC pH data logger system (Dascor, Escondido, CA). The logger was standardized by using pH 4 and 7 buffers before insertion into the rumen and right after removal from the rumen, and the pH data were recorded every minute between days 18 and 21.

### DNA extraction and bacterial and archaeal profiling by amplicon sequencing.

Total DNA was extracted from each sample (1.3 to 1.7 g/sample) by bead beating, followed by phenol-chloroform extraction (34). Each sample was adjusted to a DNA concentration of 50 ng/μl and used to amplify partial bacterial 16S rRNA gene fragments with primers A (5′-TGCTGCCTCCCGTAGGAGT-3′) and B (5′-AGAGTTTGATCCTGGCTCAG-3′) (35) and partial archaeal 16S rRNA gene fragments with Arc915aF (5′-AGGAATTGGCGGGGGAGCAC-3′) and Arc1386R (5′-GCGGTGTGTGCAAGGAGC-3′) (36, 37). All of the DNA samples were sent to Genome Quebec (Montreal, QC) for amplification of bacterial and archaeal 16S rRNA gene fragments. Briefly, bacterial 16S rRNA genes were amplified by using the thermocycler settings described by Malmuthuge et al. (38). Archaeal 16S rRNA gene fragments were amplified (50-μl reaction mixture volume) by using 50 ng of template, 5 μl of 10× buffer (Invitrogen, Carlsbad, CA), 1.5 mM MgCl_2_, 0.4 mM each primer, 10 mM each deoxynucleoside triphosphate, 0.5 U of *Taq* DNA polymerase (Invitrogen), and nuclease-free water. Amplifications were performed with an initial denaturation at 94°C for 5 min and 30 cycles of 94°C for 30 s, 58°C for 30 s, and 68°C for 1 min, followed by a final extension at 68°C for 7 min. The reaction products were run on an agarose gel, and amplicons with the proper size were trimmed and purified with the QIAquick gel extraction kit (Qiagen, ON, Canada). All of the purified amplicons were then subjected to sequencing on an Illumina MiSeq platform. Reads were processed by using Quantitative Insights into Microbial Ecology (QIIME, version 1.8.0) (39) to assess the composition of the microbial community for each sample. To obtain suitable output for functional prediction, taxonomic analyses assigned the reads to different OTUs at various taxonomic levels (phylum, family, genus, and species) on the basis of the Greengenes database (gg-13-5 version, chosen as compatible to run functional predictions) (40) and employing uclust, chimera check, and singleton removal. The species richness of each sample was estimated with the Chao1 index at 97% sequence similarity. OTUs were assigned on the basis of unique OTU reads. Shannon and Simpson indexes were calculated to indicate community diversity through QIIME. OTUs from each sample were normalized with the lowest number identified from the entire sample set prior to analysis for beta diversity (41). The relative abundance of each microbial phylotype was compared among the different levels of Tasco.

### Functional prediction of the microbial community and its association with microbial abundance.

Bacterial profiles at the species level were analyzed by using phylogenetic investigation of communities by reconstruction of unobserved states (PICRUSt, v1.1.0) to predict the metagenome of the samples (9). All of the predicted microbial functions were assigned at level 3 pathways on the basis of the Kyoto encyclopedia of genes and genomes (KEGG) database. The relative abundance of each predicted pathway was calculated, and pathways with a relative abundance of >0.01 were considered those with major functions. Pathways with a relative abundance between 0.001 and 0.01 were considered minor; and pathways with a relative abundance of <0.001 were assigned to an “others” category.

Correlations between relative microbial abundance and the predicted major functions were analyzed by using PROC CORR in SAS. Considering the small number of samples examined, only correlations with an *r* value of ≥0.5 and a *P* value of <0.001 were considered significant.

### *E. coli* serogroup identification.

To assess the presence of the seven targeted pathogenic *E. coli* O serogroups (O26, O45, O103, O111, O121, O145, and O157), samples were processed as described by Caporaso et al. (39). Briefly, a PCR mixture containing the following final concentrations of primers was prepared for each sample: O121, 50 nM; O103, O111, O145, and O157, 40 nM; O25 and O45, 25 nM. Each reaction mixture contained positive and negative controls and was used with a Verti Dx Thermal Cycler (Applied Biosystems, Burlington ON, Canada). On the basis of PCR results, immunomagnetic separation of each serogroup detected was performed by using RapidChek CONFIRM STEC kits (Romer Labs Technology Inc., Union, MO) in accordance with the manufacturer’s recommendations. Aliquots of the bead-bacterium complex (50 μl) for each target serogroup were streak plated onto MacConkey agar (MAC) with cotton swabs and incubated at 37°C for 18 to 24 h. Three to nine colonies per plate were selected, and a fraction (approximately half) of each colony was suspended in 40 μl of Tris-EDTA buffer (10 mM Tris, pH 8.0). The suspension was heated to 95°C for 5 min, and 2 μl was used as a PCR template for serogroup confirmation. The remainder of a colony confirmed positive for a target serogroup was then removed from the plate, regrown at 37°C overnight in tryptic soy broth, and stored in glycerol at −80°C. Virulence genes (encoding Shiga toxins [*stx*_1_, *stx*_2_], intimin [*eae*], and enterohemolysin [*ehxA*]) were detected by multiplex PCR with the plasmid copy number-regulating gene *repA* used as an internal control (42). Reaction mixtures containing 20 nM each primer, 1× QuantiFast master mix, nuclease-free water, and 2 μl of template DNA in a 25-μl total reaction volume were subjected to the thermocycling conditions described above. Metagenomic analysis was considered STEC positive if the *stx*_1_ or *stx*_2_ and/or *eae* genes were detected. The limit of detection was determined as described by Conrad et al. (42).

### Quantification of total bacteria, archaea, and *E. coli* by qPCR.

The total bacterial population was measured by real-time qPCR with the bacterial universal primer pair U2 (43) with initiation at 95°C for 5 min and 40 cycles of 95°C for 20 s, and 60°C for 1 min. SYBR green fluorescence signals were captured at the end of each cycle. The total archaeal population was estimated by measuring 16S rRNA gene copy numbers with archaeal universal primer pair uniMet1-F/R with a standard generated from a pure clone of *Methanobrevibacter* sp. strain AbM4 (44). The total *E. coli* population was estimated by measuring the 16S rRNA gene copy number by using *E. coli* primer pair 75F/619R with a standard generated from pure *E. coli* ATCC 25922 genomic DNA (45). The amplification program for both total archaea and total *E. coli* was as follows: initiation at 95°C for 20 s and 40 cycles of 95°C for 3 s and 60°C for 30 s, with signals captured at the end of each cycle. Melting curves were generated for all qPCR assays by using the following program: 95°C for 15 s and then 15 s at each interval with a temperature gradient of 0.3°C increments between 60°C and 95°C. SYBR green signals were captured at each increment. The melting curve of all reactions showed a single peak.

Bacterial standard generation and copy number calculations were described by Li et al. (46), and archaeal standard generation and copy number calculation were described by Zhou et al. (44). Pure genomic DNA was extracted from *E. coli* ATCC 25922 and used to generate standards to quantify *E. coli* by using the formulas of Li et al. (46). Copy numbers were estimated on the basis of plotted standard curves generated by StepOne software (V2.1; Applied Biosystems, Foster City, CA), and abundance was calculated as the number of copies per gram of content, followed by log_10_ conversion for each sample prior to further statistical analyses.

### Statistical analysis.

Microbial data were analyzed by using SAS (v9.2) and R (http://www.R-project.org). Overall microbial data were plotted by PCoA methods. The co-occurrence of microbial phylotypes was analyzed with the cooccur package within R (47). Correlation of microbial phylotypes was analyzed with the gplots package. Effects of Tasco levels (0, 1.0, 3.0, and 5.0% of the total diet DM) on microbial populations, the proportion of each phylotype, and the relative abundance of the major metabolic pathways were evaluated by using a mixed-model procedure in which a ram was the experimental unit for all of the variables tested. The general linear mixed model included the fixed effect of diet and the random effects of square, ram nested within square, and period nested in square. Sampling time was treated as a repeated measure, and denominator degrees of freedom were estimated by using Kenward-Roger approximation. In the LSMEANS statement, the PDIFF option adjusted by the Tukey method was included to enable multiple comparisons. For all comparisons, statistical significance was declared at *P* < 0.05 and trends were discussed at 0.05 ≤ *P* ≤ 0.1. Correlation analyses of microbial phylotypes and predicted functions with differential abundance among Tasco levels were conducted by using the PROC CORR model within SAS, and correlations were considered significant at *P* < 0.01.

### Availability of data.

The data set supporting the conclusions of this article is available at the NCBI SRA under BioProject ID PRJNA379293.

10.1128/mSphere.00017-18.4 FIG S4  Multiplex PCR detection of the seven *E. coli* O serogroups in individual samples. ++, positive result; blank, negative result. Download FIG S4, PDF file, 0.3 MB. Copyright © 2018 Zhou et al. 2018 Zhou et al. This content is distributed under the terms of the Creative Commons Attribution 4.0 International license.
