# Supplementary material for: Air-Dried Brown Seaweed, Ascophyllum nodosum, Alters the Rumen Microbiome in a Manner That Changes Rumen Fermentation Profiles and Lowers the Prevalence of Foodborne Pathogens
Source: mSphere. 2018 Jan 31;3(1):e00017-18. doi: 10.1128/mSphere.00017-18 (PMC5793039; doi:10.1128/mSphere.00017-18)
Supplement: TABLE S1 [file sph001182470st1.pdf]

Table S1

Proportion	Diets				Linear	Quardratic
	Con*	1SW	3SW	5SW		
Acidobacteria	0.01±0.00 [#]	0.01±0.00	0.02±0.01	0.04±0.01	NS	NS
Actinobacteria	0.99±0.18 [a]	0.76±0.10 [ab]	0.62±0.09 [ab]	0.50±0.07 [b]	0.006	0.474
Bacteroidetes	50.08±2.80	43.46±3.54	45.93±3.96	46.23±3.41	NS	NS
Chloroflexi	0.03±0.01	0.04±0.01	0.07±0.03	0.02±0.01	NS	NS
Cyanobacteria	0.10±0.03	0.24±0.08	0.24±0.04	0.34±0.11	NS	NS
Fibrobacteres	0.03±0.01	0.05±0.02	0.14±0.05	0.09±0.02	NS	NS
Firmicutes	44.04±2.95	53.55±3.59	51.76±3.94	49.43±3.69	NS	NS
Proteobacteria	3.28±1.16	1.09±0.52	0.80±0.20	2.64±1.36	NS	NS
Spirochaetes	0.17±0.04	0.24±0.07	0.10±0.03	0.16±0.05	NS	NS
Synergistetes	0.01±0.00	0.01±0.00	0.01±0.00	0.02±0.01	NS	NS
TM7	0.79±0.27 [a]	0.15±0.04 [b]	0.05±0.02 [c]	0.29±0.09 [b]	0.058	0.004
Tenericutes	0.46±0.08 [a]	0.36±0.05 [ab]	0.22±0.04 [b]	0.23±0.03 [b]	0.003	0.140

* Con: control; 1SW: 1% Tasco®; 3SW: 3% Tasco®; 5SW: 5% Tasco®.
[abc] letters indicates difference among Tasco® levels.
[#] numbers shown in percentage.
