# Supplementary material for: Air-Dried Brown Seaweed, Ascophyllum nodosum, Alters the Rumen Microbiome in a Manner That Changes Rumen Fermentation Profiles and Lowers the Prevalence of Foodborne Pathogens
Source: mSphere. 2018 Jan 31;3(1):e00017-18. doi: 10.1128/mSphere.00017-18 (PMC5793039; doi:10.1128/mSphere.00017-18)
Supplement: TABLE S2 [file sph001182470st2.pdf]

Table S2

Proportion	Diets				Linear	Quardratic
	Con*	1SW	3SW	5SW		
undefined family of Actinomycetales	0.00±0.00 [#]	0.05±0.02	0.00±0.00	0.00±0.00	NS	NS
Coriobacteriaceae	0.94±0.17 [a]	0.67±0.09 [ab]	0.56±0.09 [ab]	0.45±0.07 [b]	0.004	0.364
Bacteroidaceae	0.07±0.01	0.07±0.02	0.06±0.02	0.02±0.01	NS	NS
undefined family of class TM7-1	0.51±0.28 [a]	0.12±0.05 [b]	0.01±0.01 [c]	0.18±0.09 [b]	0.041	0.272
undefined family of class TM7-3	0.23±0.21	0.02±0.01	0.01±0.00	0.08±0.04	NS	NS
undefined family of order EW055	0.06±0.02	0.00±0.00	0.00±0.00	0.00±0.00	NS	NS
undefined family of Bacteroidales	1.16±0.40	2.14±0.62	1.53±0.70	1.90±0.77	NS	NS
Prevotellaceae	43.74±3.08	32.61±2.64	38.22±3.81	38.23±3.85	NS	NS
Rikenellaceae	0.19±0.10	0.20±0.05	0.22±0.09	0.16±0.04	NS	NS
S24_7	1.88±0.56	5.34±1.14	3.20±0.56	2.57±0.70	NS	NS
Paraprevotellaceae	2.79±0.62	3.12±0.77	2.61±0.56	2.94±0.42	NS	NS
Sphingobacteriaceae	0.22±0.06	0.34±0.09	0.05±0.01	0.36±0.14	NS	NS
Anaerolinaceae	0.03±0.01	0.03±0.01	0.07±0.03	0.02±0.01	NS	NS
undefined family of order YS2	0.00±0.00	0.14±0.12	0.08±0.04	0.22±0.14	NS	NS
undefined family of Streptophyta	0.17±0.05	0.17±0.09	0.14±0.03	0.08±0.01	NS	NS
undefined family of order 258ds10	0.00±0.00	0.06±0.03	0.14±0.07	0.09±0.03	NS	NS
Paenibacillaceae	0.22±0.07 [a]	0.08±0.03 [b]	0.22±0.08 [a]	0.97±0.39 [c]	0.007	0.069
undefined family of Clostridiales	13.18±2.69	12.29±4.17	8.99±1.37	14.99±5.44	NS	NS
Christensenellaceae	0.17±0.04	0.63±0.23	0.12±0.03	0.18±0.06	NS	NS
Clostridiaceae	0.28±0.07	0.80±0.31	0.91±0.31	1.05±0.50	NS	NS
Eubacteriaceae	0.03±0.01	0.06±0.02	0.06±0.03	0.02±0.01	NS	NS
Lachnospiraceae	13.18±1.59	12.75±1.57	11.69±2.62	8.03±0.61	NS	NS
Peptococcaceae	0.02±0.02	0.06±0.03	0.03±0.01	0.04±0.02	NS	NS
Ruminococcaceae	8.85±2.31	15.32±2.19	12.81±2.62	12.04±1.86	NS	NS
Veillonellaceae	7.83±1.27 [a]	9.04±1.58 [ab]	14.49±1.93[b]	10.25±1.48 [ab]	0.113	0.023
Mogibacteriaceae	0.41±0.10	0.49±0.08	0.54±0.11	0.37±0.06	NS	NS
Tissierellaceae	0.04±0.01	0.13±0.03	0.11±0.04	0.04±0.01	NS	NS
Erysipelotrichaceae	1.24±0.23	1.78±0.55	1.65±0.42	1.21±0.32	NS	NS
Hyphomicrobiaceae	0.01±0.00	0.08±0.05	0.03±0.02	0.01±0.00	NS	NS

undefined family of order RF39	0.46±0.13	0.39±0.06	0.22±0.05	0.22±0.03	NS	NS
Succinivibrionaceae	3.13±1.16	0.92±0.49	0.68±0.21	2.52±1.35	NS	NS
Spirochaetaceae	0.16±0.04	0.24±0.07	0.09±0.03	0.16±0.05	NS	NS

* Con: control; 1SW: 1% Tasco®; 3SW: 3% Tasco®; 5SW: 5% Tasco®.

[abc] letters indicates difference among Tasco® levels.

[#] numbers shown in percentage.
