# Supplementary material for: Air-Dried Brown Seaweed, Ascophyllum nodosum, Alters the Rumen Microbiome in a Manner That Changes Rumen Fermentation Profiles and Lowers the Prevalence of Foodborne Pathogens
Source: mSphere. 2018 Jan 31;3(1):e00017-18. doi: 10.1128/mSphere.00017-18 (PMC5793039; doi:10.1128/mSphere.00017-18)
Supplement: TABLE S3 [file sph001182470st3.pdf]

Table S3

Proportion	Diets				Linear	Quardratic
	Con*	1SW	3SW	5SW		
Prevotella	43.74±4.43 [#]	35.58±3.11	38.22±5.84	38.18±6.15	NS	NS
undefined genus of Clostridiales	11.73±4.34	20.70±11.81	8.99±1.37	14.99±5.44	NS	NS
undefined genus of Ruminococcaceae	4.36±1.18	10.26±4.23	5.74±1.62	6.92±2.56	NS	NS
Ruminococcus	4.25±3.09	9.14±3.02	6.65±3.29	3.81±1.32	NS	NS
undefined genus of Veilonellaceae	2.83±0.76	6.12±2.29	6.57±1.91	7.50±2.42	NS	NS
undefined genus of Lachnospiraceae	3.04±0.60	5.63±2.55	4.27±1.00	3.66±0.62	NS	NS
undefined genus of family S24-7	1.88±0.87	5.95±2.01	3.20±0.92	2.47±1.20	NS	NS
Dialister	2.66±1.23	3.17±1.49	5.43±2.79	0.72±0.50	NS	NS
YRC22	2.50±1.09	3.52±1.57	2.15±0.91	2.15±0.74	NS	NS
Shuttleworthia	1.19±0.95	4.18±3.07	2.79±2.70	0.30±0.11	NS	NS
undefined genus of Succinivibrionaceae	3.13±2.02	0.92±0.81	0.68±0.33	2.41±2.31	NS	NS
undefined genus of Bacteroidales	1.16±0.40	2.14±0.62	1.53±0.70	1.90±0.77	NS	NS
Butyrivibrio	1.60±0.59	1.53±0.65	1.47±0.60	0.78±0.25	NS	NS
Coprococcus	2.77±1.85	1.07±0.40	0.74±0.20	0.75±0.12	NS	NS
Roseburia	2.57±1.16 [a]	0.49±0.12 [b]	0.48±0.18 [b]	0.42±0.21 [b]	0.020	0.102
Lachnospira	0.87±0.37	0.92±0.45	0.94±0.35	0.76±0.44	NS	NS
Megasphaera	0.88±0.57	0.88±0.60	1.14±0.52	0.51±0.27	NS	NS
Succiniclasticum	0.90±0.40	0.74±0.27	0.59±0.19	1.03±0.43	NS	NS
Bulleidia	0.60±0.26	0.10±0.04	0.64±0.21	0.73±0.46	NS	NS
undefined genus of Coriobacteriaceae	0.94±0.20	0.84±0.22	0.56±0.13	0.44±0.10	NS	NS
Clostridium	0.26±0.10	0.31±0.14	0.82±0.37	0.96±0.70	NS	NS
Dorea	0.51±0.13	0.62±0.20	0.33±0.06	0.71±0.30	NS	NS
Eubacterium	0.31±0.11	0.73±0.61	0.72±0.48	0.18±0.07	NS	NS
Anaerovibrio	0.24±0.08	0.40±0.22	0.37±0.13	0.51±0.12	NS	NS
Prevotella 2	0.16±0.06	0.30±0.13	0.26±0.18	0.76±0.39	NS	NS
undefined genus of Christensenellaceae	0.16±0.05	0.95±0.56	0.11±0.04	0.18±0.08	NS	NS
undefined genus of Paenibacillaceae	0.21±0.09 [a]	0.07±0.05 [a]	0.19±0.10 [a]	0.91±0.60 [b]	0.113	0.023
Faecalibacterium	0.01±0.01	0.11±0.10	0.18±0.11	1.03±1.00	NS	NS
undefined genus of order RF39	0.46±0.13 [a]	0.39±0.06 [ab]	0.22±0.05 [b]	0.22±0.03 [b]	0.359	0.018

Mogibacterium	0.24±0.10	0.47±0.29	0.31±0.15	0.18±0.05	NS	NS
Acidaminococcus	0.31±0.12	0.29±0.11	0.36±0.15	0.19±0.10	NS	NS
Syntrophococcus	0.18±0.04 [a]	0.52±0.13 [b]	0.16±0.04 [a]	0.14±0.02 [a]	0.010	0.011
Oscillospira	0.22±0.07	0.35±0.18	0.21±0.07	0.21±0.06	NS	NS
undefined genus of Sphingobacteriaceae	0.22±0.08	0.35±0.16	0.05±0.01	0.37±0.22	NS	NS
undefined genus of Mogibacteriaceae	0.18±0.05	0.32±0.13	0.24±0.06	0.19±0.04	NS	NS
Blvii28	0.19±0.14	0.29±0.13	0.22±0.16	0.17±0.07	NS	NS
undefined genus of class TM7-1	0.51±0.28 [a]	0.12±0.05 [b]	0.01±0.01 [c]	0.18±0.09 [b]	0.041	0.272
Anaerostipes	0.20±0.10	0.31±0.18	0.11±0.05	0.13±0.09	NS	NS
Treponema	0.16±0.07	0.29±0.13	0.09±0.04	0.17±0.07	NS	NS
Blautia	0.09±0.02	0.16±0.06	0.17±0.03	0.28±0.11	NS	NS
undefined genus of Clostridiaceae	0.01±0.00	0.56±0.52	0.06±0.03	0.06±0.03	NS	NS
PSB-M-3	0.19±0.06	0.14±0.07	0.16±0.07	0.10±0.04	NS	NS
CF231	0.09±0.05	0.12±0.04	0.16±0.09	0.18±0.06	NS	NS
Ruminococcus 2	0.11±0.03	0.10±0.05	0.19±0.06	0.13±0.04	NS	NS
undefined genus of order YS2	0.02±0.01	0.14±0.12	0.08±0.04	0.22±0.14	NS	NS
undefined genus of order Streptophyta	0.07±0.03	0.17±0.09	0.14±0.03	0.08±0.01	NS	NS
undefined genus of class TM7-3	0.23±0.21	0.02±0.01	0.01±0.00	0.08±0.04	NS	NS
undefined genus of order 258ds10	0.03±0.01	0.06±0.03	0.14±0.07	0.09±0.03	NS	NS
undefined genus of Erysipelotrichaceae	0.08±0.04	0.08±0.05	0.08±0.05	0.04±0.02	NS	NS
Parvimonas	0.02±0.01	0.08±0.06	0.07±0.06	0.02±0.02	NS	NS
undefined genus of order EW055	0.06±0.02	0.00±0.00	0.00±0.00	0.00±0.00	NS	NS
Anaerolinea	0.00±0.00	0.00±0.00	0.07±0.04	0.00±0.00	NS	NS
undefined genus of Actinomycetales	0.00±0.00	0.05±0.02	0.00±0.00	0.00±0.00	NS	NS
Sharpea	0.02±0.01	0.00±0.00	0.01±0.01	0.07±0.04	NS	NS

* Con: control; 1SW: 1% Tasco®; 3SW: 3% Tasco®; 5SW: 5% Tasco®.

[abc] letters indicates difference among Tasco® levels.

[#] numbers shown in percentage.
