# Supplementary material for: Air-Dried Brown Seaweed, Ascophyllum nodosum, Alters the Rumen Microbiome in a Manner That Changes Rumen Fermentation Profiles and Lowers the Prevalence of Foodborne Pathogens
Source: mSphere. 2018 Jan 31;3(1):e00017-18. doi: 10.1128/mSphere.00017-18 (PMC5793039; doi:10.1128/mSphere.00017-18)
Supplement: TABLE S4 [file sph001182470st4.pdf]

Table S4

Proportion	Diets				Linear	Quardratic
	Con*	1SW	3SW	5SW		
**Bacteria**						
undefined sp. of Clostridiales	11.73±2.50 [#]	12.29±2.40	8.99±0.98	15.14±3.20	NS	NS
undefined sp. of Ruminococcaceae	4.6±0.76	7.14±1.11	5.74±1.03	6.89±1.51	NS	NS
Ruminococcus sp.	3.76±1.73	7.01±1.74	5.73±1.92	3.30±0.82	NS	NS
Prevotella melaninogenica	6.40±1.92	2.15±0.54	2.58±0.71	2.06±0.74	NS	NS
undefined sp. of Lachnospiraceae	3.04±0.40	3.90±0.79	4.27±0.60	3.54±0.35	NS	NS
undefined sp. of family S24-7	1.88±0.56	5.34±1.14	3.20±0.56	2.57±0.70	NS	NS
Dialister sp.	2.66±0.73	2.70±0.85	5.43±1.56	0.75±0.29	NS	NS
undefined sp. of genera YRC22	2.50±0.60	2.62±0.71	2.15±0.56	1.97±0.41	NS	NS
Shuttleworthia sp.	1.19±0.59	4.16±1.73	2.79±1.66	0.29±0.08	NS	NS
undefined sp. of Succinivibrionaceae	3.13±1.16	0.92±0.49	0.68±0.21	2.52±1.35	NS	NS
undefined sp. of Bacteroidales	1.16±0.23	1.71±0.25	1.53±0.40	1.89±0.45	NS	NS
Butyrivibrio sp.	1.60±0.43	1.04±0.15	1.47±0.35	0.79±0.16	NS	NS
Lachnospira sp.	0.87±0.21	0.91±0.26	0.94±0.22	0.80±0.25	NS	NS
Megasphaera sp.	0.88±0.35	0.87±0.39	1.14±0.35	0.53±0.18	NS	NS
*Succiniclasticum*sp.	0.90±0.26	0.55±0.10	0.59±0.12	1.00±0.27	NS	NS
Ruminococcus flavefaciens	0.49±0.08	0.82±0.18	0.92±0.18	0.56±0.07	NS	NS
Bulleidia sp.	0.60±0.18	0.77±0.23	0.61±0.13	0.75±0.28	NS	NS
Clostridium sp.	0.26±0.07	0.20±0.04	0.82±0.28	0.97±0.48	NS	NS
Dorea sp.	0.49±0.09	0.55±0.12	0.33±0.04	0.70±0.19	NS	NS
Eubacterium dolichum	0.31±0.08	0.73±0.34	0.72±0.27	0.19±0.04	NS	NS
Prevotella sp. 2	0.16±0.04	0.30±0.07	0.26±0.11	0.76±0.26	NS	NS
Anaerovibrio sp.	0.24±0.06	0.25±0.05	0.37±0.08	0.50±0.08	NS	NS
undefined sp. of Paenibacillaceae	0.21±0.07	0.07±0.03	0.19±0.07	0.95±0.39	NS	NS
Faecalibacterium prausnitzii	0.01±0.00	0.11±0.09	0.18±0.07	1.08±0.59	NS	NS
Acidaminococcus sp.	0.31±0.08	0.29±0.09	0.36±0.10	0.20±0.06	NS	NS
undefined sp. of Christensenellaceae	0.16±0.04	0.62±0.23	0.11±0.03	0.18±0.06	NS	NS
undefined sp. of Sphingobacteriaceae	0.22±0.06	0.34±0.09	0.05±0.01	0.36±0.13	NS	NS

Mogibacterium sp.	0.24±0.07	0.27±0.06	0.31±0.09	0.18±0.03	NS	NS
Syntrophococcus sucromutans	0.18±0.03	0.42±0.07	0.16±0.03	0.14±0.02	NS	NS
Oscillospira sp.	0.22±0.05	0.22±0.05	0.21±0.05	0.21±0.04	NS	NS
undefined sp. of Mogibacteriaceae	0.18±0.04	0.22±0.03	0.24±0.04	0.19±0.03	NS	NS
undefined sp. of class TM7-1	0.51±0.20	0.11±0.04	0.01±0.00	0.19±0.06	NS	NS
undefined sp. of genera Blvii28	0.19±0.10	0.20±0.05	0.22±0.09	0.16±0.04	NS	NS
Anaerostipes sp.	0.20±0.06	0.31±0.10	0.11±0.03	0.14±0.05	NS	NS
undefined sp. of Clostridiaceae	0.01±0.00	0.55±0.29	0.06±0.02	0.06±0.02	NS	NS
Treponema sp.	0.16±0.04	0.24±0.07	0.09±0.03	0.16±0.05	NS	NS
undefined sp. of order YS2	0.02±0.01	0.14±0.08	0.08±0.03	0.23±0.11	NS	NS
undefined sp. of genera CF231	0.09±0.01	0.12±0.02	0.16±0.02	0.25±0.06	NS	NS
undefined sp. of genera PSB-M-3	0.19±0.04	0.14±0.06	0.16±0.05	0.11±0.03	NS	NS
Parvimonas sp.	0.02±0.01	0.07±0.03	0.07±0.03	0.02±0.01	NS	NS
Ruminococcus gnavus	0.11±0.03	0.09±0.03	0.19±0.05	0.13±0.03	NS	NS
undefined sp. of genera Blvii28	0.13±0.03	0.06±0.02	0.11±0.04	0.09±0.03	NS	NS
undefined sp. of Streptophyta	0.07±0.02	0.10±0.03	0.14±0.03	0.08±0.02	NS	NS
undefined sp. of order 258ds10	0.03±0.01	0.05±0.02	0.14±0.05	0.09±0.02	NS	NS
undefined sp. of class TM7-3	0.23±0.14	0.02±0.01	0.01±0.00	0.08±0.03	NS	NS
undefined sp. o Erysipelotrichaceae	0.08±0.03	0.05±0.01	0.08±0.03	0.05±0.01	NS	NS
Anaerolinea sp.	0.03±0.01	0.03±0.01	0.07±0.03	0.02±0.01	NS	NS
Eubacterium sp.	0.03±0.01	0.06±0.02	0.06±0.03	0.02±0.00	NS	NS
undefined sp. of order EW055	0.06±0.02	0.02±0.01	0.03±0.02	0.02±0.01	NS	NS
undefined sp. of genera 5-7N15	0.05±0.01	0.04±0.01	0.04±0.02	0.01±0.00	NS	NS
Moryella sp.	0.02±0.01	0.02±0.01	0.04±0.01	0.03±0.01	NS	NS
Paraprevotella sp.	0.03±0.01	0.04±0.01	0.03±0.01	0.02±0.01	NS	NS
undefined sp. of Hyphomicrobiaceae	0.00±0.00	0.08±0.04	0.03±0.02	0.00±0.00	NS	NS
Sharpea sp.	0.02±0.01	0.01±0.00	0.01±0.00	0.08±0.03	NS	NS
undefined sp. of Comamonadaceae	0.00±0.00	0.03±0.01	0.01±0.01	0.04±0.02	NS	NS
undefined sp. of Peptococcaceae	0.02±0.01	0.03±0.01	0.02±0.01	0.03±0.01	NS	NS
Bulleidia p-1630-c5	0.01±0.00	0.04±0.01	0.03±0.01	0.00±0.00	NS	NS
Bacteroides sp.	0.02±0.01	0.03±0.01	0.01±0.00	0.01±0.01	NS	NS
Tissierella sp.	0.02±0.01	0.05±0.02	0.02±0.01	0.00±0.00	NS	NS

undefined sp. of Actinomycetales	0.02±0.01	0.04±0.01	0.02±0.01	0.02±0.01	NS	NS
undefined sp. of Bacillales	0.02±0.01	0.02±0.01	0.01±0.01	0.04±0.01	NS	NS
undefined sp. of Paraprevotellaceae	0.00±0.00	0.06±0.03	0.02±0.01	0.00±0.00	NS	NS
Ethanoligenens sp.	0.01±0.00	0.02±0.01	0.05±0.02	0.01±0.01	NS	NS
Dorea formicigenerans	0.03±0.02	0.02±0.01	0.00±0.00	0.02±0.01	NS	NS
undefined sp. of genera p-75-a5	0.02±0.01	0.01±0.00	0.02±0.01	0.02±0.01	NS	NS
undefined sp. of Pirellulaceae	0.01±0.01	0.02±0.01	0.01±0.00	0.02±0.01	NS	NS
undefined sp. of Alphaproteobacteria	0.03±0.02	0.01±0.00	0.02±0.01	0.00±0.00	NS	NS
undefined sp. of Acidimicrobiales	0.01±0.00	0.02±0.01	0.02±0.01	0.01±0.00	NS	NS
Erysipelothrix	0.00±0.00	0.03±0.01	0.02±0.01	0.01±0.00	NS	NS
Selenomonas sp.	0.00±0.00	0.01±0.00	0.02±0.01	0.03±0.02	NS	NS
Paenibacillus sp.	0.01±0.00	0.01±0.00	0.02±0.01	0.02±0.01	NS	NS
Prevotella sp. 1	0.01±0.00	0.02±0.01	0.01±0.00	0.02±0.01	NS	NS
Oribacterium sp.	0.01±0.01	0.01±0.01	0.01±0.01	0.01±0.01	NS	NS
Gracilibacter sp.	0.01±0.01	0.03±0.02	0.01±0.01	0.00±0.00	NS	NS
undefined sp. of order CCU21	0.00±0.00	0.01±0.00	0.01±0.00	0.03±0.01	NS	NS
undefined sp. of Acidaminobacteraceae	0.00±0.00	0.02±0.01	0.01±0.01	0.01±0.00	NS	NS
undefined sp. of genera SMB53	0.00±0.00	0.02±0.01	0.01±0.00	0.00±0.00	NS	NS
undefined sp. of family RF16	0.01±0.00	0.02±0.01	0.00±0.00	0.00±0.00	NS	NS
Veillonella sp.	0.01±0.00	0.01±0.00	0.01±0.01	0.01±0.01	NS	NS
Parabacteroides sp.	0.00±0.00	0.00±0.00	0.02±0.01	0.01±0.01	NS	NS
undefined sp. of Alicylobacillaceae	0.01±0.01	0.01±0.00	0.01±0.00	0.01±0.00	NS	NS
Tindallia sp.	0.00±0.00	0.01±0.00	0.02±0.01	0.01±0.00	NS	NS
undefined sp. of Stramenopiles	0.00±0.00	0.00±0.00	0.01±0.00	0.02±0.01	NS	NS
Bifidobacterium sp.	0.01±0.01	0.01±0.00	0.01±0.00	0.01±0.00	NS	NS
Blautia sp.	0.01±0.00	0.00±0.00	0.01±0.00	0.02±0.01	NS	NS
Alicyclobacillus sp.	0.00±0.00	0.02±0.01	0.01±0.01	0.00±0.00	NS	NS
Christensenella sp.	0.00±0.00	0.01±0.01	0.01±0.00	0.01±0.00	NS	NS
undefined sp. of Microthrixaceae	0.00±0.00	0.01±0.01	0.01±0.00	0.01±0.00	NS	NS
Lactobacillus sp.	0.00±0.00	0.01±0.00	0.02±0.02	0.00±0.00	NS	NS
undefined sp. of Sva0725	0.00±0.00	0.00±0.00	0.01±0.01	0.01±0.01	NS	NS
undefined sp. of genera HA73	0.00±0.00	0.01±0.00	0.01±0.00	0.01±0.01	NS	NS

undefined sp. of genera TG5	0.01±0.00	0.00±0.00	0.01±0.00	0.00±0.00	NS	NS
Desulfobulbus sp.	0.00±0.00	0.01±0.00	0.01±0.01	0.01±0.01	NS	NS
Pedobacteri sp.	0.00±0.00	0.00±0.00	0.01±0.00	0.01±0.00	NS	NS
Dehalobacterium sp.	0.00±0.00	0.01±0.00	0.01±0.01	0.00±0.00	NS	NS
Peptococcus sp.	0.00±0.00	0.00±0.00	0.01±0.00	0.01±0.01	NS	NS
Sphaerochaeta sp.	0.01±0.01	0.01±0.00	0.00±0.00	0.00±0.00	NS	NS
Coprobacillus	0.01±0.00	0.01±0.01	0.00±0.00	0.01±0.01	NS	NS
Fusobacterium sp.	0.00±0.00	0.00±0.00	0.02±0.01	0.00±0.00	NS	NS
Alkaliphilus sp.	0.00±0.00	0.01±0.00	0.00±0.00	0.01±0.01	NS	NS
Tepidimicrobium sp.	0.00±0.00	0.00±0.00	0.01±0.01	0.00±0.00	NS	NS
**Archaea**						
Methanobrevibacter gottschalkii	44.14±5.12	39.52±4.11	39.10±5.03	40.24±3.46	NS	NS
Methanobrevibacter ruminantium	9.94±2.04	22.56±3.76	15.84±3.61	13.40±2.77	NS	NS
Methanobrevibacter wolinii	12.07±4.59	5.65±2.85	7.11±3.84	12.61±4.89	NS	NS
Methanosphaera sp.	33.18±3.17	25.86±4.19	36.68±3.65	27.60±2.64	NS	NS
undefined sp. of Methanopasmatales	0.67±0.28	6.39±3.06	1.26±0.51	6.14±2.08	NS	NS
**Protozoa**						
Polyplastron sp.	9.76±2.29	13.84±3.59	13.23±3.55	19.87±2.59	NS	NS
Isotricha sp. 1	2.01±0.90	3.03±1.32	1.35±0.48	3.71±1.23	NS	NS
Isotricha sp. 2	69.1±7.08	58.85±10.47	57.28±11.16	38.66±7.96	NS	NS

* Con: control; 1SW: 1% Tasco®; 3SW: 3% Tasco®; 5SW: 5% Tasco®.

^{abc} letters indicates difference among Tasco® levels.

$^{\#}$ numbers shown in percentage.
