# Supplementary figures and images for: Air-Dried Brown Seaweed, Ascophyllum nodosum, Alters the Rumen Microbiome in a Manner That Changes Rumen Fermentation Profiles and Lowers the Prevalence of Foodborne Pathogens
Source: mSphere. 2018 Jan 31;3(1):e00017-18. doi: 10.1128/mSphere.00017-18 (PMC5793039; doi:10.1128/mSphere.00017-18)

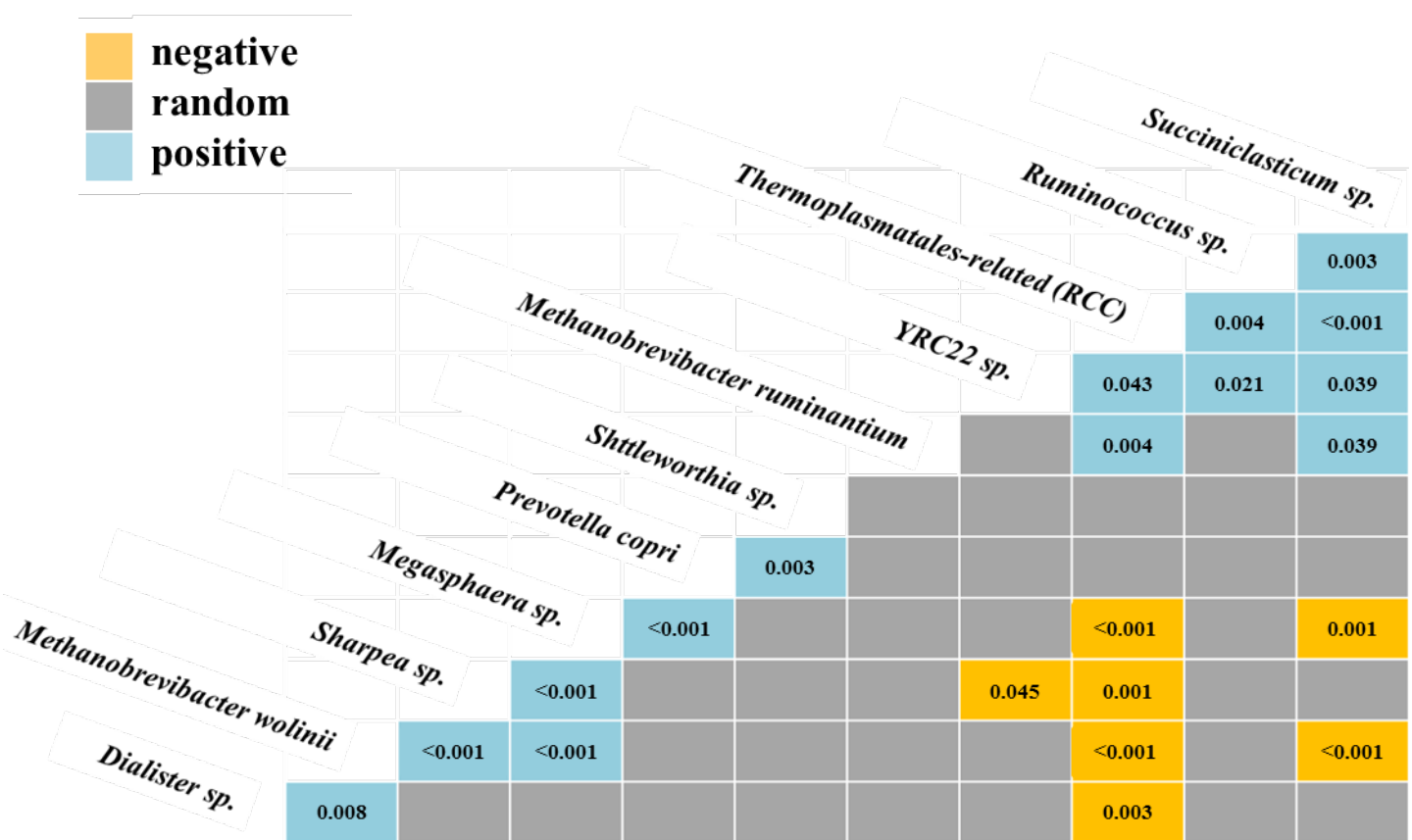

Supplement: FIG S1 [file sph001182470sf1.pdf]
