# Supplementary material for: Air-Dried Brown Seaweed, Ascophyllum nodosum, Alters the Rumen Microbiome in a Manner That Changes Rumen Fermentation Profiles and Lowers the Prevalence of Foodborne Pathogens
Source: mSphere. 2018 Jan 31;3(1):e00017-18. doi: 10.1128/mSphere.00017-18 (PMC5793039; doi:10.1128/mSphere.00017-18)
Supplement: FIG S2 [file sph001182470sf2.pdf]

	Acetic	Propionic	Isobutyric	Butyric	Isovaleric	Valeric	Caproic	Acetic: Propionic	NH_3-N
**Undefined sp. of Vellonellaceae**							**0.314**		
Prevotella copri						**0.308**			
***Roseburia* sp.**		**0.312**							
Blautia producta						**0.348**			
***Entodinium* sp. 1**	**0.429**							**0.387**	**0.498**
***Polyplastron* sp.**	**0.489**							**0.461**	**0.550**
***Isotricha* sp. 1**	**0.329**							**0.357**	**0.310**
***Isotricha* sp. 2**		**0.428**				**0.538**			

Zhou et al., Figure S2
