# Supplementary material for: Air-Dried Brown Seaweed, Ascophyllum nodosum, Alters the Rumen Microbiome in a Manner That Changes Rumen Fermentation Profiles and Lowers the Prevalence of Foodborne Pathogens
Source: mSphere. 2018 Jan 31;3(1):e00017-18. doi: 10.1128/mSphere.00017-18 (PMC5793039; doi:10.1128/mSphere.00017-18)
Supplement: FIG S3 [file sph001182470sf3.pdf]

	acetic	propionic	isobutyric	butyric	isovaleric	valeric	caproic
Acidobacteria	0.084	0.350	0.610	0.255	0.577	0.129	0.058
Actinobacteria	0.419	0.885	0.380	0.488	0.209	0.722	0.365
Bacteroidetes	0.009	<0.001	0.407	0.121	0.923	0.068	0.108
Chloroflexi	0.011	0.001	0.744	0.186	0.729	0.012	0.962
Cyanobacteria	0.679	0.189	0.402	0.276	0.302	0.325	0.954
Fibrobacteres	0.213	<0.001	0.026	0.002	0.992	0.001	0.435
Firmicutes	<0.001	<0.001	0.870	0.106	0.302	0.027	0.067
Proteobacteria	0.004	<0.001	0.658	0.251	<0.001	0.031	0.316
Spirochaetes	0.707	<0.001	<0.001	<0.001	0.092	<0.001	0.293
Synergistetes	0.977	0.375	0.345	0.386	0.603	0.470	0.311
TM7	0.476	0.321	0.019	0.070	0.167	0.370	0.531
Tenericutes	0.018	0.992	0.155	0.024	0.150	0.350	0.597

-0.50 0.50
