# Supplementary material for: Air-Dried Brown Seaweed, Ascophyllum nodosum, Alters the Rumen Microbiome in a Manner That Changes Rumen Fermentation Profiles and Lowers the Prevalence of Foodborne Pathogens
Source: mSphere. 2018 Jan 31;3(1):e00017-18. doi: 10.1128/mSphere.00017-18 (PMC5793039; doi:10.1128/mSphere.00017-18)
Supplement: TABLE S5 [file sph001182470st5.pdf]

Table S5

Microbial phylotype	Function	Correlation coefficient	P
**Bacterial family**			
Coriobacteriaceae	Tyrosine metabolism	0.4177	<0.001
	Fructose and mannose metabolism	0.4779	<0.001
	Glycolysis / Gluconeogenesis	0.4194	<0.001
	Pentose phosphate pathway	0.4812	<0.001
	Propanoate metabolism	0.4224	<0.001
	Pyruvate metabolism	0.3970	<0.001
	Starch and sucrose metabolism	0.2172	0.035
	Carbon fixation in photosynthetic organisms	0.2997	0.003
	Methane metabolism	0.2257	0.028
	Fatty acid biosynthesis	0.3266	0.001
	D-Alanine metabolism	0.2681	0.009
	Biosynthesis of ansamycins	0.3284	0.001
	Tetracycline biosynthesis	0.3923	<0.001
	Benzoate degradation	0.4397	<0.001
	Chloroalkane and chloroalkene degradation	0.4059	<0.001
	Naphthalene degradation	0.3311	0.001
	Polycyclic aromatic hydrocarbon degradation	0.2638	0.001
Paenibacillaceae	Glycolysis / Gluconeogenesis	0.2100	0.041
Veillonellaceae	Amino acid related enzymes	0.4153	<0.001
	Lysine degradation	0.2491	0.015
	Valine leucine and isoleucine degradation	0.3490	<0.001
	Novobiocin biosynthesis	0.3552	<0.001
	Tropane piperidine and pyridine alkaloid biosynthesis	0.2924	0.004
**Bacterial genus**			
Roseburia	Phenylpropanoid biosynthesis	0.3854	<0.001
	Glyoxylate and dicarboxylate metabolism	0.2371	0.021
	Starch and sucrose metabolism	0.3474	<0.001
	Photosynthesis	0.4501	<0.001
	Photosynthesis proteins	0.4358	<0.001
	Sulfur metabolism	0.5142	<0.001
	Glycerophospholipid metabolism	0.5878	<0.001
	Biotin metabolism	0.3576	<0.001
	Porphyrin and chlorophyll metabolism	0.3808	<0.001
	Riboflavin metabolism	0.2096	0.042
	Thiamine metabolism	0.5958	<0.001

	Cyanoamino acid metabolism	0.3104	0.002
	Selenocompound metabolism	0.4166	<0.001
	Biosynthesis of ansamycins	0.4243	<0.001
	Glutamatergic synapse	0.7122	<0.001
undefined genus of Paenibacillaceae	Glycolysis / Gluconeogenesis	0.2109	0.040
undefined genus of order RF39	Streptomycin biosynthesis	0.2294	0.025
	Amino sugar and nucleotide sugar metabolism	0.2154	0.036
	Polyketide sugar unit biosynthesis	0.2614	0.011
**Archaeal species**			
undefined sp. of Methanopasmatales	Lysine degradation	0.3558	<0.001
	Phenylpropanoid biosynthesis	-0.2665	0.009
	Butanoate metabolism	0.3069	0.003
