# Supplementary material for: Air-Dried Brown Seaweed, Ascophyllum nodosum, Alters the Rumen Microbiome in a Manner That Changes Rumen Fermentation Profiles and Lowers the Prevalence of Foodborne Pathogens
Source: mSphere. 2018 Jan 31;3(1):e00017-18. doi: 10.1128/mSphere.00017-18 (PMC5793039; doi:10.1128/mSphere.00017-18)
Supplement: TABLE S6 [file sph001182470st6.pdf]

Table S6[a]

Species 1	Species 2	sp1-inc*	sp2-inc	obs-cooccur	prob-cooccur	exp-cooccur	p-lt	p-gt
O26_ru	O157_ru	2	24	1	0.047	1.5	0.44	0.94
O45_ru	O103_ru	12	12	6	0.141	4.5	0.93	0.22
O45_ru	O111_ru	12	12	4	0.141	4.5	0.50	0.77
O45_ru	O121_ru	12	7	5	0.082	2.6	0.99	0.05
O45_ru	O145_ru	12	6	2	0.070	2.2	0.60	0.75
O45_ru	O157_ru	12	24	11	0.281	9	0.99	0.10
O45_ru	O26_fe	12	4	2	0.047	1.5	0.86	0.48
O45_ru	O45_fe	12	4	3	0.047	1.5	0.99	0.14
O45_ru	O121_fe	12	3	1	0.035	1.1	0.69	0.77
O103_ru	O111_ru	12	12	7	0.141	4.5	0.99	0.07
O103_ru	O121_ru	12	7	4	0.082	2.6	0.95	0.22
O103_ru	O145_ru	12	6	1	0.070	2.2	0.25	0.96
O103_ru	O157_ru	12	24	9	0.281	9	0.66	0.67
O103_ru	O26_fe	12	4	1	0.047	1.5	0.52	0.87
O103_ru	O45_fe	12	4	1	0.047	1.5	0.52	0.87
O103_ru	O121_fe	12	3	0	0.035	1.1	0.23	1.00
O111_ru	O121_ru	12	7	4	0.082	2.6	0.95	0.22
O111_ru	O145_ru	12	6	2	0.070	2.2	0.60	0.75
O111_ru	O157_ru	12	24	9	0.281	9	0.66	0.67
O111_ru	O26_fe	12	4	3	0.047	1.5	0.99	0.14
O111_ru	O45_fe	12	4	2	0.047	1.5	0.86	0.48
O111_ru	O121_fe	12	3	0	0.035	1.1	0.23	1.00
O121_ru	O145_ru	7	6	0	0.041	1.3	0.20	1.00
O121_ru	O157_ru	7	24	6	0.164	5.2	0.90	0.42
O145_ru	O157_ru	6	24	6	0.141	4.5	1.00	0.15
O157_ru	O26_fe	24	4	4	0.094	3	1.00	0.30
O157_ru	O45_fe	24	4	3	0.094	3	0.70	0.75
O157_ru	O103_fe	24	2	2	0.047	1.5	1.00	0.56
O157_ru	O111_fe	24	2	2	0.047	1.5	1.00	0.56
O157_ru	O121_fe	24	3	3	0.070	2.2	1.00	0.41

[a] Co-occurrence coefficient and probability would only be calculated by the program when O-serotype being observed for at least 2 times in one site while more than 2 times in the other site.
* sp1-inc: Number of sites (or samples) that have species 1; sp2-inc: Number of sites that have species 2; obs-cooccur: Observed number of sites having both species; prob-cooccur: Probability that both species occur at a site; exp-cooccur: Expected number of sites having both species; p-lt: Probability that the two species would co-occur at a frequency less than the observed number of co-occurrence sites if the two species were distributed randomly (independently) of one another; p-gt: Probability of co-occurrence at a frequency greater than the observed frequency.
