# Supplementary material for: Air-Dried Brown Seaweed, Ascophyllum nodosum, Alters the Rumen Microbiome in a Manner That Changes Rumen Fermentation Profiles and Lowers the Prevalence of Foodborne Pathogens
Source: mSphere. 2018 Jan 31;3(1):e00017-18. doi: 10.1128/mSphere.00017-18 (PMC5793039; doi:10.1128/mSphere.00017-18)
Supplement: FIG S4 [file sph001182470sf4.pdf]

		Rumen							Feces						
**BASELINE**	**07-January-2014**			**serogroup**								**serogroup**			
Ram	Diet	O26	O45	O103	O111	O121	O145	O157	O26	O45	O103	O111	O121	O145	O157
1	control				++	++						++			
2	control		++	++						++	++		++		++
3	control						++					++			
4	control	++			++			++	++						
5	control		++					++	++			++	++		
6	control			++					++			++	++		
7	control			++		++	++	++					++		
8	control			++			++	++	++			++	++	++	
**PERIOD 1**	**28-January-2014**			**serogroup**								**serogroup**			
Ram	Diet	O26	O45	O103	O111	O121	O145	O157	O26	O45	O103	O111	O121	O145	O157
1	control						++	++				++	++		
2	1%-SW							++							
3	5%-SW														
4	1%-SW				++			++	++	++		++			
5	3%-SW			++	++			++							
6	3%-SW				++										
7	control			++				++							
8	5%-SW			++	++										
**PERIOD 2**	**18-February-2014**			**serogroup**								**serogroup**			
Ram	Diet	O26	O45	O103	O111	O121	O145	O157	O26	O45	O103	O111	O121	O145	O157
1	1%-SW		++					++							
2	5%-SW														
3	3%-SW							++							
4	5%-SW														
5	control			++											
6	control							++							
7	1%-SW					++									
8	3%-SW		++	++				++							
**PERIOD 3**	**11-March-2014**			**serogroup**								**serogroup**			
Ram	Diet	O26	O45	O103	O111	O121	O145	O157	O26	O45	O103	O111	O121	O145	O157
1	3%-SW		++					++							
2	control	++	++	++	++					++					
3	1%-SW		++	++	++	++		++							
4	control		++			++		++		++					
5	5%-SW		++	++	++	++		++							
6	5%-SW	++	++		++	++		++	++						
7	3%-SW		++	++		++		++							
8	1%-SW		++					++	++		++				
**PERIOD 4**	**01-April-2014**			**serogroup**								**serogroup**			
Ram	Diet	O26	O45	O103	O111	O121	O145	O157	O26	O45	O103	O111	O121	O145	O157
1	5%-SW		++	++			++	++							
2	3%-SW		++	++	++		++	++							
3	control				++		++	++							
4	3%-SW			++	++			++							
5	1%-SW						++	++					++		
6	1%-SW				++		++	++							
7	5%-SW							++							
8	control		++				++	++		++	++		++		

Zhou et al., Figure S4
